# Strategies for Improving Selectivity and Sensitivity of Schiff Base Fluorescent Chemosensors for Toxic and Heavy Metals

**DOI:** 10.3390/molecules28196960

**Published:** 2023-10-06

**Authors:** Brian Musikavanhu, Yongdi Liang, Zhaoli Xue, Lei Feng, Long Zhao

**Affiliations:** 1 School of Chemistry and Chemical Engineering, Jiangsu University, Zhenjiang 212013, China; brianmusikavanhu@gmail.com (B.M.); 2222212020@stmail.ujs.edu.cn (Y.L.); zhaolixue@ujs.edu.cn (Z.X.); 2 Monash Suzhou Research Institute, Monash University, Suzhou Industrial Park, Suzhou 215000, China; leifeng@monash.edu

**Keywords:** Schiff base, chemosensor, functional groups, nanomaterials, heavy metal cations

## Abstract

Toxic cations, including heavy metals, pose significant environmental and health risks, necessitating the development of reliable detection methods. This review investigates the techniques and approaches used to strengthen the sensitivity and selectivity of Schiff base fluorescent chemosensors designed specifically to detect toxic and heavy metal cations. The paper explores a range of strategies, including functional group variations, structural modifications, and the integration of nanomaterials or auxiliary receptors, to amplify the efficiency of these chemosensors. By improving selectivity towards targeted cations and achieving heightened sensitivity and detection limits, consequently, these strategies contribute to the advancement of accurate and efficient detection methods while increasing the range of end-use applications. The findings discussed in this review offer valuable insights into the potential of leveraging Schiff base fluorescent chemosensors for the accurate and reliable detection and monitoring of heavy metal cations in various fields, including environmental monitoring, biomedical research, and industrial safety.

## 1. Introduction

Schiff bases are a class of organic compounds characterized by an imine (-C=N-) functional group formed by the reaction between the amine amino group and the aldehyde or ketone carbonyl group [1]. This imine functionality imparts important chemical and biological properties to Schiff bases, endowing diverse applications of these molecules in various fields of chemistry, to coordinate and complexate with metal ions. Due to the presence of the imine group, Schiff base compounds can act as ligands, forming transition metal Schiff base complexes [2,3]. These complexes often exhibit valuable properties, such as catalytic activity, fluorescence, and magnetic behavior. In addition to their coordination chemistry, Schiff bases also find applications in organic synthesis and medicinal chemistry [4,5]. They can be used as intermediates in preparing various organic compounds and are commonly employed in synthesizing dyes, pigments, and pharmaceuticals. Schiff base compounds have also shown various biological activities, including antimicrobial [6], antifungal [7], anticancer [8], and antioxidant properties [9].

The synthesis of Schiff bases involves the condensation reaction between any combination of aliphatic or aromatic amines and aldehydes/ketones with a reactive carbonyl group. While the procedure is generally the same, the specific conditions, such as reactant ratio, solvent, temperature, and reaction time, may vary depending on the reactants and desired product. In general, the amine and the aldehyde/ketone are mixed in a suitable solvent, such as ethanol or methanol. The ratio of reactants is typically 1:1, but it can be slightly modified to obtain a larger yield of the desired product [10]. Acidic or basic catalysts, depending on the reaction conditions, can be used to promote the condensation reaction. The reaction mixture is heated or refluxed for a specific time, which also varies based on the reactants and conditions. The reaction can be monitored using techniques such as thin-layer chromatography, or other suitable methods. The disappearance of the starting materials and the appearance of the desired product often indicate the completion of the reaction. The mixture is cooled once the reaction is complete, and the Schiff base product is then isolated. This can be done through filtration, crystallization, or extraction techniques to separate the desired compound from any impurities. Spectroscopic techniques such as infrared (IR) spectroscopy, nuclear magnetic resonance (NMR) spectroscopy, and mass spectrometry are often employed to analyze the structure [11,12,13].

Quite a few review papers have been dedicated to the field of chemosensors for heavy metal ions detections in environmental and biological applications in recent years [14,15,16,17,18,19,20,21,22]. Some of them have focused on a single major fluorophore building block to track different ions. For instance, Mansha et al. reviewed coumarin-derived chemosensors, focusing on the sensing approach and efficiency, while Pooja et al. discussed their pH limit, detection limit, and binding mode for different ions [23,24]. Choudhury et al. discussed polymeric sensors bearing pendant rhodamine units, focusing on how their high-aqueous solubility, improved stability, signal amplification, and easy fabrication into devices make them worth exploring [25]. Sarkar et al. also discussed rhodamine dye-based sensor molecules, but they focused on binding affinity, limit of detection (LOD) of some functionalized rhodamine-based chemosensors that emit in the near-infrared region (NIR) [26]. Muhammad et al. delved into thiourea and its derivatives, fixating on structural parameters and coordination mechanism of each sensor towards different metal ions [27]. Others have focused on specific metal ions. Alharbi at al. explored the performance of different organic compounds, including Schiff bases, thiourea, pyridine, and rhodamine, as chemosensors specifically for Zn^2+^ [16]. Trevino et al. reviewed small-molecule Cu^2+^ sensors that provide a color response visible to the naked eye in solutions [28]. Here, the focus was on the molecular structural features and sensing mechanisms in environmental and biological samples. Macrocycle-based molecules as chemosensors for lead, cadmium, and mercury ions were discussed by Zhang et al. [29]. They discussed in detail how conformational flexibility, cavity size, and the number and disposition of donor atoms can influence performance.

To be specific, this review aims to summarize the current state of research and provide an overview of the existing literature on Schiff base fluorescent chemosensors for toxic and heavy metal cations from 2018 to 2023. It focuses on their unique molecular structure, synthesis methods, and properties, which differ from other chemosensors. It delves into the underlying sensing mechanisms, outlining their unique interactions with the target analytes. The diverse applications are explored, including detection and monitoring of various analytes such as metal ions and environmental pollutants. We analyze various strategies, including functional group variations, structural modifications, and integrating nanomaterials or auxiliary receptors to amplify these chemosensors’ efficiency. It will analyze the limitations of existing chemosensors by critically evaluating the selectivity and sensitivity of Schiff base fluorescent chemosensors for toxic and heavy metal cations. Challenges and shortcomings current chemosensors face regarding cross-reactivity with other metal ions, LOD, or interference from complex sample matrices will also be addressed. Additionally, this review will shed light on emerging trends and provide a glimpse into the future by identifying recent advancements and potential directions. It will explore novel sensing mechanisms, innovative designs, and advanced analytical methods that hold the potential to enhance the selectivity and sensitivity of these chemosensors. By addressing these aspects, this review aims to contribute to the advancement of the field by providing valuable insights, recommendations, and guidance for the development of more effective Schiff base fluorescent chemosensors for the detection of toxic cations.

## 2. Structural Features and Chemistry of Schiff Bases

Schiff bases have couples of important structural features and exhibit diverse properties, endowing them with important compounds in coordination chemistry, organic synthesis, and medicinal chemistry. Their ability to form coordination complexes and reactivity opens opportunities for their application in various fields. Some key aspects include:

(1) Imine Functional Group: The defining structural feature of Schiff bases is the presence of an imine (-C=N-) functional group. This double bond between carbon and nitrogen is formed through the condensation reaction between a primary amine and an aldehyde or ketone. The imine group imparts unique chemical and physical properties to Schiff bases, making them versatile compounds [30].
(2) Chelation: Schiff bases can act as ligands, forming coordination complexes with metal ions. The imine nitrogen atom can donate its lone pair of electrons to form a coordination bond with a metal ion, forming Schiff base metal complexes. This chelation capability enables the use of Schiff bases in various fields such as catalysis, materials science, and coordination chemistry [31].
(3) Tautomeric Forms: Schiff bases can exist in different tautomeric forms, primarily the imine and enamine tautomers. The imine form has a double bond between the carbon and nitrogen atoms, while the enamine form has a carbon–carbon double bond adjacent to the imine nitrogen. The equilibrium between these tautomeric forms can influence the reactivity and properties of Schiff bases [32].
(4) Acid-Base Properties: Schiff bases can act as both acids and bases. The imine nitrogen can participate in protonation reactions, making the Schiff base compound acidic. On the other hand, the nitrogen lone pair can accept protons, making Schiff bases basic. Schiff bases’ acidic and basic characteristics contribute to their reactivity and coordination ability [33].
(5) Reactivity: Schiff bases exhibit rich reactivity due to the presence of the imine group. They can undergo chemical transformations such as hydrolysis, reduction, oxidation, and nucleophilic addition reactions. The reactivity of Schiff bases makes them valuable intermediates for the synthesis of diverse organic compounds and coordination complexes [34].
(6) Biological Activity: Schiff bases have shown a range of biological activities and applications. They have been studied for their antimicrobial and antifungal activity, such as Schiff base derivatives of quinoline, anticancer by interfering with cancer cell growth and inducing apoptosis, and antioxidant properties in neutralizing harmful free radicals in the body. Schiff base complexes with transition metal ions have been widely applied in various biological areas, such as metal ion detection, catalysis, and medicinal chemistry [35,36].

In addition, Schiff bases exhibit interesting photophysical features that make them attractive for various applications, including optoelectronics, sensor technology, and molecular electronics. These properties make Schiff bases versatile and promising materials for various photonic and optoelectronic applications. Some key photophysical properties of Schiff bases include:

(1) Absorption and Emission Ability: Schiff bases typically have broad absorption and emission spectra, meaning that they can absorb light at a specific wavelength and emit light at a longer wavelength [37]. The absorption spectra cover a wide range of wavelengths, allowing them to absorb light in the visible or near-ultraviolet region. Such optical behavior of Schiff bases is highly dependent on their molecular structure and electronic environment. By modifying the structure of Schiff bases, their fluorescence properties can be tuned, leading to the development of fluorescent probes and sensors [38].
(2) Solvent and pH Sensitivity: The photophysical properties of Schiff bases can be influenced by the surrounding medium’s polarity and acidity/basicity. Solvent polarity or pH changes can alter Schiff bases’ absorption and emission wavelengths, quantum yields, and fluorescence lifetimes. This sensitivity to the environment makes them useful for sensing applications [39].
(3) Nonlinear Optical Properties: Schiff bases with extended π-conjugation can possess nonlinear optical properties. They exhibit efficient nonlinear optical responses such as second harmonic generation and third-order frequency mixing, making them potential candidates for applications in nonlinear optics, photonic devices, and optical data storage [40,41].

## 3. Schiff Bases as Fluorescent Chemosensors for Hazardous Ions

Schiff bases have been extensively explored as fluorescent chemosensors for detecting and monitoring hazardous cations. These cations include heavy metal ions such as mercury (Hg^2+^), lead (Pb^2+^), cadmium (Cd^2+^), and other toxic metal ions that pose significant environmental and health risks [42]. These chemosensors offer significant advantages such as real-time monitoring, high sensitivity, and selectivity, making them invaluable tools in environmental monitoring, biomedical diagnostics, and industrial quality control. However, to meet the ever-growing demands for improved performance, researchers continuously explore novel strategies to enhance the selectivity and sensitivity of Schiff base chemosensors towards toxic cations [43]. The following are some of the ways that Schiff bases function as fluorescent chemosensors for hazardous cations:

(1) Selective Binding: Schiff bases can be designed to exhibit high selectivity and affinity for specific hazardous cations. The imine moiety in Schiff bases can act as a binding site for these cations, facilitating coordination through lone pair–electron interactions. By incorporating specific functional groups or structural modifications, Schiff bases can be tuned to selectively recognize and bind specific hazardous cations, even in the presence of other metal ions [44].
(2) Fluorescence Quenching Mechanism: Schiff bases with inherent fluorescence properties can undergo fluorescence quenching upon binding to hazardous cations. The coordination between the Schiff base’s cation and the imine group disrupts the fluorescent properties. This quenching effect can arise due to energy transfer, electron transfer, or other quenching mechanisms associated with coordination with the hazardous cation [45].
(3) Fluorescence Recovery: The fluorescence quenching of the Schiff base upon cation binding provides the basis for sensing hazardous cations. The fluorescence quenching occurs when the Schiff base is exposed to a hazardous cation sample. The presence and concentration of the cation can be determined by monitoring the fluorescence recovery upon chelation or displacement of the hazardous cation [46].
(4) Sensitivity and LOD: Schiff bases can exhibit high sensitivity towards hazardous cations, allowing for their detection at low concentrations. The extent of fluorescence quenching or recovery correlates with the concentration of the hazardous cation in the sample, which allows for quantitative detection within a specific range. The detection limits can be optimized by modifying the Schiff base structure and carefully selecting the fluorescent properties [47].
(5) Sensor Design: The design of chemosensors for hazardous cations is critical to achieving high selectivity and sensitivity. Factors such as the choice of Schiff base structure, the nature of the coordinating groups, and the optimal fluorophore functionality are considered to enhance the sensing properties. This design approach ensures minimal interference from other metal ions and improved analytical performance [48].
(6) Real-Time Monitoring: The reversible binding of hazardous cations to Schiff bases enables real-time monitoring of cation concentrations. This is particularly valuable for continuous monitoring or in-field measurements of hazardous cations in environmental, industrial, or biological settings [49].

Using Schiff bases as fluorescent chemosensors for hazardous cations offers a promising approach for rapid, selective, and sensitive detection. Their ability to undergo fluorescence quenching and recovery upon interaction with the target cations allows for accurate monitoring and assessment of the presence and concentration of hazardous metal ions. Beyond the measurements, understanding the sensing mechanisms behind the phenomena is also an important scientific issue. Here are some common sensing mechanisms of Schiff base chemosensors:

(1) Chelation-Induced Fluorescence Modulation (CHEF): Schiff base chemosensors can exhibit chelation-induced fluorescence modulation. The formation of a chelate complex between the Schiff base and the analyte induces conformational changes, altering the molecular environment around the fluorophore. These changes can affect the fluorophore’s excited state and result in fluorescence intensity, emission wavelength, or fluorescence lifetime changes [50].
(2) Protonation/Deprotonation: Schiff bases can act as pH-responsive chemosensors. Changes in pH alter the protonation/deprotonation state of the Schiff base, leading to changes in the electronic environment and fluorescence properties. Protonation or deprotonation can induce changes in the chromophore’s electronic structure, such as charge redistribution or intramolecular charge transfer (ICT), resulting in fluorescence changes that can be measured [51].
(3) Photoinduced Electron Transfer (PET): Schiff base chemosensors can exhibit fluorescence changes due to photoinduced electron transfer processes. This mechanism involves electron transfer between the analyte and the excited state of the Schiff base. The analyte’s electron-withdrawing or electron-donating properties can influence fluorescence, leading to fluorescence quenching or enhancement [52].
(4) Excited-State Intramolecular Proton Transfer (ESIPT): Some Schiff bases undergo ESIPT processes, where upon excitation, a proton migrates intramolecularly from the hydroxyl or amino group of the Schiff base to an adjacent electron-rich group. This results in a significant change in the fluorescence emission wavelength. ESIPT can lead to dual-emission and ratiometric sensing capabilities, making Schiff bases valuable for fluorescence-based pH sensors [53].
(5) Aggregation-Induced Emission (AIE): AIE in Schiff base compounds refers to the phenomenon where these compounds exhibit enhanced fluorescence intensity upon aggregation or confinement. When Schiff base compounds undergo aggregation or are confined within specific environments, their molecular motions become restricted, and non-radiative energy pathways that typically cause fluorescence quenching are blocked. As a result, the fluorescence emission of Schiff base compounds with AIE characteristics is significantly increased, leading to bright fluorescence signals. This unique behavior has attracted significant attention and has been extensively explored for various applications [54,55,56].
(6) Förster Resonance Energy Transfer (FRET): Schiff base chemosensors can utilize FRET as a sensing mechanism. FRET occurs when the excited-state energy of the Schiff base donor fluorophore is transferred to an acceptor fluorophore upon analyte binding. The analyte-induced proximity between the donor and acceptor leads to a decrease or increase in fluorescence intensity, depending on the specific design and energy transfer efficiency [57].

These sensing mechanisms of Schiff base chemosensors enable the detection and monitoring of various analytes, including cations, anions, pH, small molecules, and biomolecules. By understanding and leveraging these mechanisms, Schiff base chemosensors can be designed to exhibit high selectivity, sensitivity, and responsiveness, making them valuable tools in various analytical, environmental, and biomedical applications.

## 4. Modifications and Variations in Functional Groups

One of the key strategies employed involves variations in functional groups within the Schiff base structure [58,59]. By modifying the aromatic rings, substituents, or linker groups, researchers have successfully fine-tuned the properties of the chemosensors to target specific toxic cations selectively. For instance, the introduction of electron-withdrawing groups such as nitro or cyano moieties has been shown to enhance the affinity towards metal cations by increasing the electron density on the Schiff base system [60,61]. On the other hand, electron-donating groups such as amino or hydroxyl groups have been utilized to improve the sensitivity towards softer toxic cations. Other ways include optimizing the spatial arrangement and rigidity of the receptor portion to facilitate efficient binding and recognition of toxic cations. For instance, introducing bulky substituents or chelating moieties on the receptor platform has enhanced selectivity towards specific metal cations [62]. 

### 4.1. Schiff Bases as Chemosensors for Hg^2+^

Mercury can accumulate in certain fish and seafood species, posing a risk to ecosystems and human health, particularly pregnant women and young children [50]. Chronic mercury exposure can lead to serious health problems, including neurological and developmental disorders. Chemosensors for mercury can be used to detect and monitor the levels of mercury in air, water, and soil. By developing sensitive and selective chemosensors, we can better understand the distribution and concentration of mercury, identify pollution sources, and take necessary measures to reduce its impact on the environment [63,64]. In addition, chemosensors can be employed to assess mercury levels in food products, ensuring compliance with safety regulations and providing consumers with information to make informed decisions about their dietary choices.

**Figure 1:**
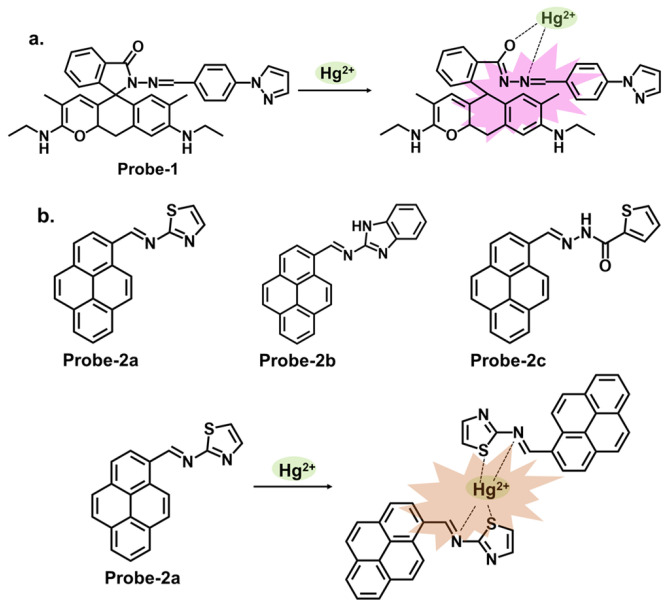
The structures and mechanisms of pyrazole and pyrene-derived Schiff base chemosensors for Hg^2+^; (**a**) **probe-1**, and (**b**) **probes-2a**, **2b**, and **2c**.

One way of ensuring that chemosensors realize their full potential is by incorporating specific functional groups that can work in synergy to influence the physical and chemical properties of the sensor. Pyrazole derivatives can be used as Schiff base chemosensors because of their versatility. The pyrazole ring consists of a five-membered aromatic ring containing three carbon atoms and two adjacent nitrogen atoms, as shown in Figure 1a [65]. The pyrazole itself is non-fluorescent, but when incorporated into other moieties, the compound’s electronic properties will be altered. These changes can be detected through various spectroscopic techniques, such as UV–vis absorption spectroscopy, fluorescence spectroscopy, or electrochemical methods. The sensing mechanism of pyrazole Schiff base chemosensors can involve various interactions, such as coordination bonding, hydrogen bonding, or π–π stacking. These interactions can induce changes in the spectroscopic properties of the compound, allowing for the detection and quantification of the target analyte. An example of this is a report by Yang et al., who prepared a Schiff base probe (**probe-1**) for detecting Hg^2+^ in aqueous solutions by linking rhodamine 6G with a pyrazole derivative [66]. The methodology was clearly outlined, which enhances reproducibility. This unique combination of moieties produced a functioning probe that is weakly fluorescent due to the C=N isomerization. Adding Hg^2+^ produced a new π-conjugated structure, leading to a turn-on reaction and a distinct color change from colorless to pink. The turn-on reaction was determined to result from an inhibited PET mechanism, leading to the CHEF process. Sensibility was quite high, with an LOD value estimated at 0.2 nM, which compares well with other comparable probes (Table 1). For application, the probe was successfully used on paper strips as a calorimetric sensor to track trace amounts of Hg^2+^ from water. The study, however, relies heavily on spectral changes and neglected to comprehensively evaluate other important parameters, such as stability, solvent effects and response time, which could provide a reference for other researchers pursuing similar structures. Another example where functional groups can induce changes in the electronic properties is a series of pyrene-based Schiff base Hg^2+^ trackers **probes-2a**, **2b**, and **2c**, shown in Figure 1b [67]. It sheds light on the performance of pyrene-based compounds as fluorescent chemosensors, and several critical aspects outlined in this study warrant discussion. As expected, the different functional groups produced different performance results. Upon performing a series of analytical techniques, the probe containing thiophene-2-carbohydrazide (**2a**) performed the best, recording an LOD of 0.27 μM. It exhibited high selectivity and anti-interference properties when tested in other ions. However, while the selectivity towards Hg^2+^ and the subsequent turn-on response was attributed to the hard and soft acid-base (HSAB) theory and hydrolysis of the imine (C=N) bond, the underlying mechanistic insights into the fluorescence activation upon binding could be explored further to enrich the understanding of the sensor’s behavior. The absence of data on stability and solvent effect also makes it difficult to ascertain the overall performance of this probe.

**Figure 2:**
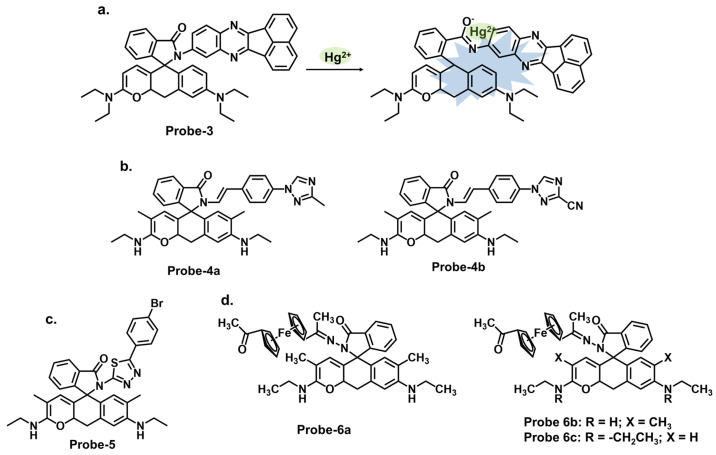
The structures (and mechanisms) of rhodamine-based Schiff base chemosensors for Hg^2+^; (**a**) **probe-3**, (**b**) **probes-4a**, **4b**, (**c**) **probe-5**, and (**d**) **probes-6a**, **6b**, **6c**.

Rhodamine is a fluorescent dye that can be used as a chemosensor in applications such as detecting metal ions, pH changes, and biological molecules. Rhodamine-based chemosensors work by undergoing a change in fluorescence intensity or color in the presence of specific analytes. It commonly exists in equilibrium between two forms. The open form is the fluorescent conformation and is dominant in acidic conditions. The closed form is the non-fluorescent spirolactone form, as illustrated in Figure 2. One example is **probe-3** studied by Cui et al., which is a Schiff base receptor based on rhodamine-b for tracking Hg^2+^ [68]. The use of rhodamine is commendable since it is well known for its fluorescence enhancement properties. As expected, the turn-on reception resulted from the opening of the spirolactam ring once Hg^2+^ was added. Various tests showed the probe to be highly selective and sensitive, with an LOD value of 10 nM. Here, however, further investigation on the high selectivity towards Hg^2+^ compared to other soft acids such as Cd^2+^ and Ag^+^ would provide a deeper understanding of the underlying physical phenomena beyond the ring opening. The probe was successfully used for imaging live HeLa cells and zebrafish, further proving to be a valuable contribution to in vivo applications. Table 1 outlines how probe-3 compares to other sensors in terms of LOD, mechanism, and application. **Probes-4a** and **4b** are Schiff base receptors also bearing a rhodamine backbone reported by Zhong et al., where they incorporated triazolyl benzaldehyde moieties to the rhodamine 6G fluorophore to create sensors capable of tracking Hg^2+^ [69]. The study systematically explores the performance of the probes via a series of experiments, including UV–vis, emission studies, and metal ion titration. UV–vis and fluorescence investigations showed the probes linked with Hg^2+^ in a 1:1 ratio with high selectivity and sensitivity. The LOD values were also very low, estimated at 13.4 nM and 15.6 nM, respectively. The turn-on response was determined to result from the suppression of PET and C=N isomerization once the complex was formed. For application, the probes were successfully used in MCF-7 cells. While there are a lot of interesting findings, the lack of a comprehensive discussion of other rhodamine-based probes with similar mechanisms and their limitations makes it difficult to evaluate the superiority of this probe. Vanjare et al. synthesized **probe-5**, a rhodamine 6 G-based Schiff base probe for tracking Hg^2+^ in the nanomolar range [70]. The probe’s response towards Hg^2+^ and other ions is presented with clarified calorimetric and fluorescence changes. The probe’s closed spirocyclic form is non-fluorescent. When Hg^2+^ is added, it forms a 1:1 complex, resulting in the opening of the spiro ring and a corresponding enhanced fluorescence. Interference from other ions was minimal, and the sensitivity was quite high, with the LOD estimated at 30.37 nM. The probe also efficiently recovered Hg^2+^ from different water sources at rates above 96%. Cell imaging was also successfully performed in MDA-MB-231 and A375 cells with very low cytotoxicity. While the spectral changes have been comprehensively explored, a study, however, on potential limitations, including photobleaching, side reactions, or other degradation, would be helpful to enhance the scientific rigor and provide more reference points to other researchers seeking to optimize similar structures. Dewangan et al. synthesized unsymmetrical ferrocene-based organometallic Schiff base probes by incorporating ferrocenyl species into rhodamine moieties (**probes-6a**, **6b**, and **6c**) [71]. A detailed description of the synthesis and purification, as well as the analytical methods employed, is provided, which boosts reproducibility. The probes have poor emission but adding Hg^2+^ results in enhanced fluorescence and a corresponding increase in quantum yield. Various tests in solution and THP-1 cancer cells showed that the probes can be used to track Hg^2+^ in solution and at the cellular level. However, detailed comparisons with other similar structures are lacking, which hinders the assessment of its superiority or equivalency in terms of precision and accuracy.

**Figure 3:**
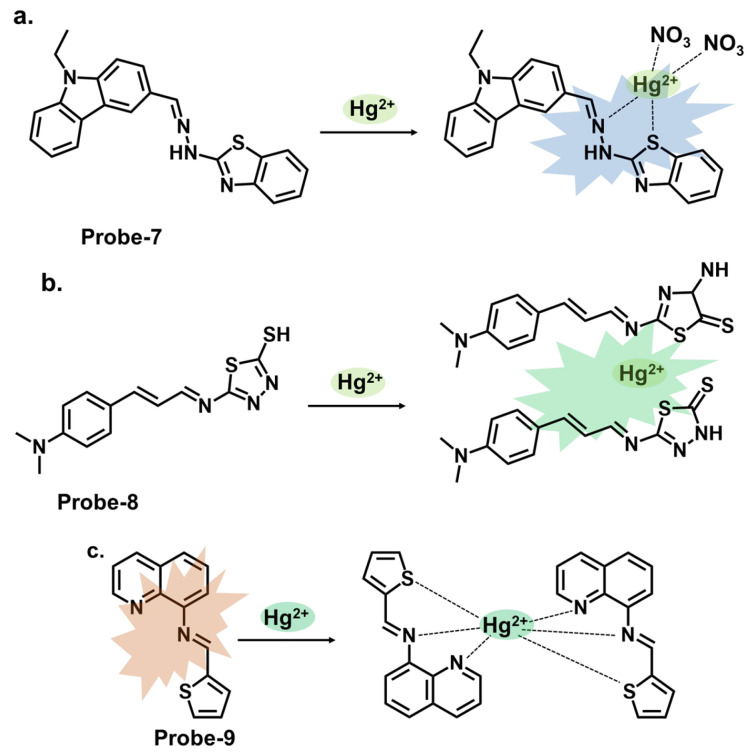
The structures and mechanisms of carbazole-, thiadiazole-, and quinoline-based Schiff base chemosensors for Hg^2+^; (**a**) **probe-7**, (**b**) **probe-8**, and (**c**) **probe-9**.

Carbazole is a tricyclic aromatic compound that exhibits fluorescence properties. Carbazole-based chemosensors can be designed and synthesized to detect and respond to specific analytes, such as metal ions, organic molecules, or environmental pollutants. These chemosensors work by changing fluorescence intensity, emission wavelength, or other spectroscopic properties upon interaction with the target analyte. The specific design strategies and applications of carbazole-based chemosensors can vary depending on the desired sensing objective. Leslee et al. reported **probe-7**, shown in Figure 3a, a Schiff base receptor with carbazole and benzothiazole moieties, for the calorimetric and fluorimetric detection of Hg^2+^ ions [72]. The turn-on sensor is weakly fluorescent because of the ICT process between the carbazole unit and benzothiazole moiety. Fluorescence is enhanced upon complexation with Hg^2+^ ions, with an estimated LOD of 0.14 μM. The experiments performed clearly illustrate the enhancement mechanism and establish a correlation between the concentration of Hg^2+^ ions and fluorescence response. The analytical techniques were performed and confirmed the probe to be immune to interference and that the coordination occurred between the Hg^2+^ ions and the nitrogen and sulfur atoms of the hydrazine and benzothiazole units, respectively. This affinity towards Hg^2+^ was attributed to the HSAB principle, though there was no deep dive into why other soft acids such as Ag^+^ and Cd^2+^ did not have the same effect. Furthermore, the 3D spectral analysis showed that the probe could be viable for tracking Hg^2+^ ions in cells and cellular organelles. However, the study could benefit from response time analysis to ascertain its potential application in real-time monitoring. Reversibility studies would also provide further insight into how it responds to strong chelating agents like EDTA.

Similarly, thiadiazole is a heterocyclic compound containing sulfur and nitrogen atoms that has been incorporated into different sensor designs. The specific design strategies and applications of these sensors can vary depending on the desired sensing objective. Singh et al. reported **probe-8**, shown in Figure 3b, which is a dual Schiff base sensor for the turn-on detection of Hg^2+^ and turn-off detection of Ag^+^ ions [73]. **Probe-8** was synthesized in methanol in a one-step reaction between 4-(dimethylamino)cinnamaldehyde and 5-amino-1,3,4-thiadiazole-2-thiol. The synergy between the different moieties allowed the probe to have a high affinity for Hg^2+^ with very low interference. The turn-on detection of Hg^2+^ was possibly due to CHEF, with an estimated LOD value of 2 µM. In contrast, the turn-off response towards Ag^+^ was possibly due to aggregation-caused quenching (ACQ). While the spectral changes were clearly explained, a nuanced analysis of the underlying physical phenomena, in this case CHEF and ACQ, would enhance our understanding of this dual-mechanism probe. Data on solvent effects and overall stability would also give some ideas about its limitations. Quinoline and thiophene are two other heterocyclic organic compounds, whose derivatives have applications in the synthesis of dyes and fluorescent probes. Our group reported a quinoline–thiophene Schiff base sensor (**probe-9**) for tracking Hg^2+^ (Figure 3c) [50]. The probe underwent chelation-enhanced quenching due to the coordination between Hg^2+^ and the sulfur and nitrogen atoms. The LOD was estimated at 23 nM, while Job’s plot confirmed a 2:1 binding ratio. The HSAB theory was used to explain the high selectivity towards Hg^2+^. **Probe-9** was also successfully used to track Hg^2+^ in HeLa cells. While the pertinent parameters were investigated and discussed, a more comprehensive comparison with other structures and a detailed outline of the possible limitations could provide an even better understanding of this sensor in terms of practicality and reliability. 

### 4.2. Schiff Bases as Chemosensors for Cu^2+^

Copper is widely used in various industries, including electronics, construction, and manufacturing. Monitoring copper levels in industrial processes is important to ensure compliance with regulations and prevent the release of copper into the environment. Chemosensors for copper contribute to scientific research and education by providing tools for studying copper’s behavior, transport, and interactions in various environments. They can aid in understanding copper’s role in biogeochemical cycles, the impact of copper pollution on ecosystems, and the development of remediation strategies.

**Figure 4:**
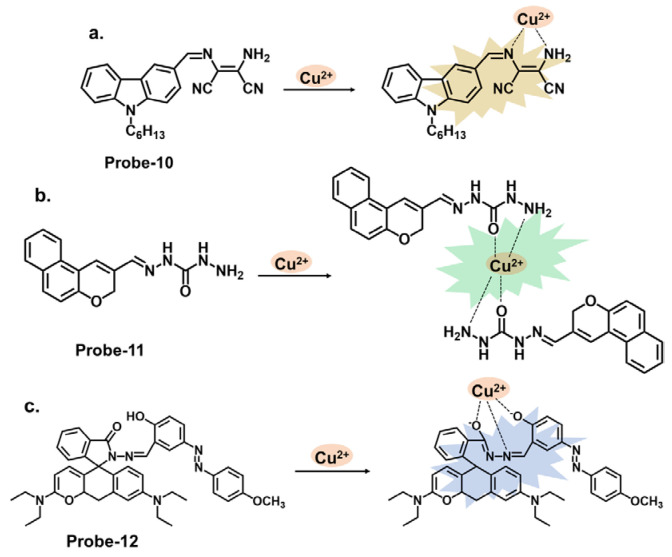
The structures and mechanisms of carbazole-, carbazide-, and rhodamine-based Schiff base chemosensors for Cu^2+^; (**a**) **probe-10**, (**b**) **probe-11**, and (**c**) **probe-12**.

Carbazole derivatives can be designed to specifically target Cu^2+^. The principle is the same as those to Hg^2+^, where changes in color, fluorescence intensity, emission wavelength, or other spectroscopic properties are observed upon interaction with Cu^2+^. A prime example is 2-amino-3-((Z)-((9-hexyl-9H-carbazol-3-yl) methylene) amino) maleonitrile (**probe-10**), shown in Figure 4a, reported by Yin et al., which is a carbazole-derived Schiff base probe prepared in a three-step process to detect Cu^2+^ [74]. **Probe-10** is weakly fluorescent due to the active C=N isomerism. Upon adding Cu^2+^, it exhibited a color change from yellow to colorless and a 160-fold fluorescence enhancement with high selectivity and minimal interference. Job’s plot determined a 1:1 binding ratio, while UV–vis titration estimated the LOD at 27.4 nM. The binding was also shown to be reversible by adding EDTA. While the spectral analyses were thoroughly performed and appear to show negligible interference and marked selectivity towards Cu^2+^, the underlying reasons for this could be explored further to make it easier to predict and tune behavior in complex environments. Unlike carbazoles, monocarbazones consist of one benzene ring instead of two, as shown in Figure 4b. They can be used in synergy with other functional groups to create sensors that target specific analytes. **Probe-11** is a monocarbazone Schiff base Cu^2+^ tracker derived from 2*H*-benzo[*h*]chromene-3-carbaldehyde and carbazide by Wang et al. [75]. Changes in fluorescence intensity often indicate the only detection signal in most probes so this ratiometric sensor is interesting. **Probe-11**, which is AIE-active, showed enhanced emission in Cu^2+^ due to a mechanism involving intermolecular charge transfer coupled with a metal-induced assembly. Its calorimetric behavior was confirmed by the color change from brown to colorless. Job’s plot confirmed the 1:1 binding ratio, while further analysis estimated the LOD to be 10.4 nM, respectively. Additionally, the probe was successfully used to track Cu^2+^ ions in HeLa cells. **Probe-11** proved highly sensitive, reversible, and immune to interference from many other cations. It would be interesting, however, to gain an understanding of its limitations, specifically on photostability, which has been known to affect longevity and performance. **Probe-12**, shown in Figure 4c, is a rhodamine derivative synthesized by Yang et al. to detect Cu^2+^ and cysteine [76]. The relevant experiments were performed and support the claims made by the authors. A complex is formed upon introducing Cu^2+^, which opens the lactam ring, resulting in enhanced emission intensity and increased quantum yield. This turn-on reaction was not observed when other divalent and trivalent ions were introduced. **Probe-12** was also used to detect Cu^2+^ in living HepG2 cells and Kunming mice with negligible cytotoxicity. Rhodamine derivatives have been explored extensively in the field of fluorescence chemosensors, and a comprehensive comparison with other similar structures would make it easier to assess **probe-12**, whether in terms of dynamic range or response time, and make modifications if necessary.

**Figure 5:**
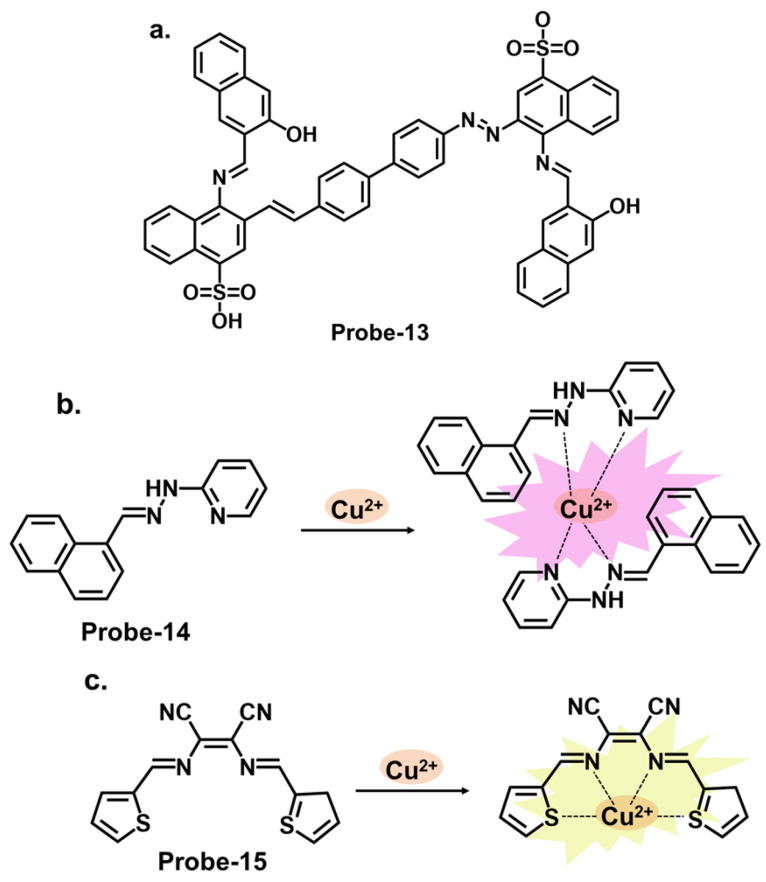
The structures (and mechanisms) of benzidine-, pyridine-, and thiophene-derived Schiff base chemosensors for Cu^2+^; (**a**) **probe-13**, (**b**) **probe-14**, and (**c**) **probe-15**.

Benzidine, pyridine, and naphthalene groups, shown in Figure 5a–c, respectively, have been incorporated into various fluorescence probes to track Cu^2+^ cation. Congo red is a benzidine-based dye that is commonly used as a biological stain, particularly for amyloid fibrils. It has also been explored as a fluorescent chemosensor for detecting various analytes, including metal ions and biological molecules. Ni et al. reported **probe-13**, a Schiff base Cu^2+^ tracker synthesized via a one-step process by refluxing 1-hydroxynaphthalene-2-carbaldehyde and Congo red in ethanol [77]. The methodology offers a detailed account of the synthesis process and the characterization techniques. In Cu^2+^, **probe-13** showed colorimetric and fluorescence turn-on responses, traced to the inhibition of C=N isomerism and CHEF. This was accompanied by a color change from brown to colorless, while other cations did not cause any emission or color changes. Job’s plot confirmed a 1:1 stoichiometry, while other analyses estimated the LOD to be 0.1 nM. As illustrated in Table 1, this LOD is amongst the lowest in the reviewed probes. Furthermore, **probe-13** showed low cytotoxicity and good cell membrane permeability when tested in HepG2 cells. While the key performance indicators were sufficiently explored, assessing the influence of temperature, pH, and solvent polarity on the sensing abilities would contribute to a more comprehensive understanding of its practical utility and durability. Xiao et al. reported **probe-14**, a naphthalene- and pyridine-bearing Schiff base Cu^2+^ tracker synthesized from the reaction between 2-naphthaldehyde and 2-hydrazinylpyridine [78]. The structure is simple, but several critical aspects, reversibility being one of them, warrant discussion. The probe is weakly fluorescent because PET from the lone pairs on nitrogen to the naphthalene moiety quenches the fluorescence. When Cu^2+^ forms a complex with **probe-14**, PET is inhibited, resulting in enhanced emission, which is the basis of the detection process. Job’s plot confirmed a 2:1 binding ratio, with an LOD estimated at 3.90 nM. It was also responsive towards the S^2−^ ion, as the anion could displace the Cu^2+^ ion from the complex. While the LOD is sufficiently low to reversibly track Cu^2+^ ions in the nanomolar range, an investigation into the response time would clarify whether it can be used for real-time monitoring. **Probe-15** reported by Venkatesan et al. is a Schiff base chemosensor for detecting Cu^2+^, synthesized by the reaction between thiophene carbaldehyde and diamino malononitrile in methanol [79]. The fluorogenic sensor changes color from yellow to colorless in Cu^2+^, with a 1:1 binding ratio and an LOD of 14.5 nM. **Probe-15** exhibited anti-interference properties and high selectivity towards Cu^2+^ only. The turn-on response was attributed to the CHEF mechanism upon forming the **probe-15-**Cu^2+^ complex. It was also observed to be reversible when tested with a powerful chelating agent like EDTA. For practical applications, **probe-15** was successfully used to recover Cu^2+^ in various water samples. This study presents a systematic investigation into the important parameters and the authors’ claims aligned with the obtained data. For this probe, conducting experiments under diverse environmental conditions would strengthen its suitability for real-world scenarios. Investigations on temperature stability and solvent compatibility are particularly important to assess practical applicability.

**Figure 6:**
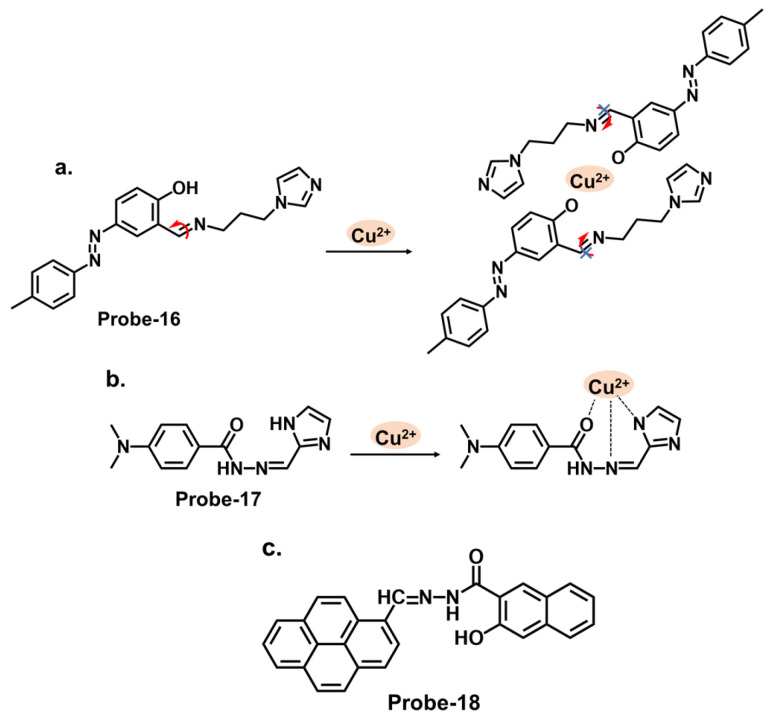
The structures (and mechanisms) of imidazole- and pyrene-based Schiff base chemosensors for Cu^2+^; (**a**) **probe-16**, (**b**) **probe-17**, and (**c**) **probe-18**.

Some successful Schiff base chemosensors for Cu^2+^ detection are derived from imidazole derivatives. One example is **probe-16**, shown in Figure 6a, which is synthesized by Slassi et al. from the reaction between N-(3-Aminopropyl) imidazole and 2-Hydroxy-5-(p-tolyldiazenyl) benzaldehyde [80]. **Probe-16** is weakly fluorescent because of the active C=N isomerism within the structure. In the presence of Cu^2+^, the **probe-16-**Cu^2+^ complex is formed, which restricts C=N isomerism, resulting in CHEF. In this structure, the imidazole moiety is the electron donor, while the hydroxyl and the imine groups form the binding sites. Job’s plot determined the binding ratio to be 2:1, while the detection limit was estimated at 1.8 μM. The range of analytes used to assess selectivity could be expanded to eliminate the chances of other ions being false positives during application. Another example is **probe-17**, shown in Figure 6b, which is a colorimetric and fluorescent Schiff base sensor for detecting Cu^2+^, synthesized by refluxing 4-Dimethylamino-benzohydrazide and imidazole-2-formaldehyde [81]. NMR spectra and mass spectroscopy confirmed a successful synthesis. This probe had a turn-on reaction towards Cu^2+^, with a color change from colorless to yellow and an LOD of 15 nM. Job’s plot confirmed a 1:1 binding ratio, while reversibility was tested with S^2−^, which could displace Cu^2+^ from the complex. For application, **probe-17** could show distinct color changes when used on paper strips. It recovered Cu^2+^ from various water sources and blood, with recovery rates above 97%. While the sensing mechanism was thoroughly investigated through experimental and theoretical studies, more in-depth discussion on the obtained data, juxtaposed with other similar structures, would provide deeper insights into the underlying physical phenomena that lead to this turn-on response. More chemosensor examples from cyclic compounds with relatively high sensitivity and selectivity for Cu^2+^ are also reviewed. Pyrenes are polycyclic hydrocarbons consisting of four fused benzene rings, which are typically used as building blocks for the construction of fluorescence chemosensors (Figure 6c). Arjunan et al. reported **probe-18**, a pyrene motif Schiff base Cu^2+^ tracker synthesized by refluxing 1-pyrene carboxaldehyde and 3-hydroxy-2-napthoic hydrazide in ethanol [82]. **Probe-18** is weakly fluorescent because of active PET processes. PET is inhibited upon coordination with Cu^2+^, resulting in a turn-on response. While the response time was relatively long (5 min), the LOD was quite low (0.26 μM), indicating generally high sensitivity. Furthermore, the probe detected Cu^2+^ ions in living RAW 264.7 cells with very low cytotoxicity. Although the laboratory-based experiments have been carried out meticulously, the potential real-world applications of the sensor could be investigated further, as the practical utility of the chemosensor is crucial for its broader acceptance and adoption.

**Figure 7:**
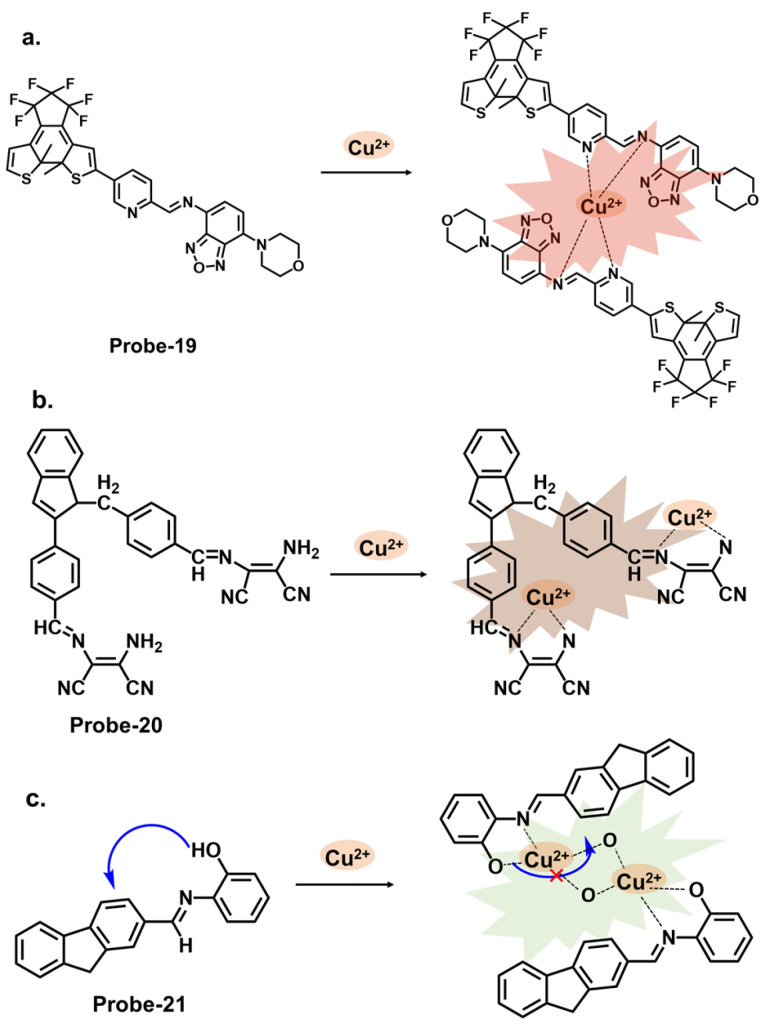
The structures and mechanisms of diarylethene-, benzimidazole-, and fluorene-derived Schiff base chemosensors for Cu^2+^; (**a**) **probe-19**, (**b**) **probe-20**, and (**c**) **probe-21**.

Diarylethene compounds are noted for their characteristic reversible photochromism, which makes them valuable in optical data storage, molecular switches, and fluorescent sensors. The fluorescence of a diarylethene molecule can be controlled by modulating its molecular conformation through external stimuli, such as light or analyte interactions. Gao et al. reported **probe-19**, shown in Figure 7a, which is a Schiff base receptor based on a diarylethene with benzo [1,2,5] oxadiazol-4-ylamine, prepared in a two-step reaction to track Cu^2+^ [83]. **Probe-19** detects Cu^2+^ in a turn-on reaction with a 90-fold increase in emission intensity, accompanied by a color change from dark red to bright red. The enhanced emission response was due to the suppression of the C=N isomerization, which led to the CHEF process. Job’s plot confirmed a 2:1 binding ratio, while UV–vis titration estimated the LOD at 1.49 μM. Further analyses showed very little interference and high selectivity. For application, it was used to recover Cu^2+^ from river water, with recovery rates above 90%. This sensor showed promising results, and further investigations into the stability and photostability under various environmental conditions, such as pH and temperature variations, would provide valuable insights for other potential practical applications. Two more examples are shown in Figure 7. **Probe-20**, shown in Figure 7b, is a benzimidazole-based Schiff base probe with a bidentate ligand for tracking Cu^2+^ [84]. It is weakly fluorescent, but a turn-on response was observed when Cu^2+^ was added. This enhanced emission was attributed to the inhibition of C=N isomerization and PET process between the diaminomaleonitrile group and the benzimidazole unit. Job’s plot, mass spectra, and DFT analysis confirmed a 1:2 binding ratio, while UV–vis titration estimated the LOD to be 0.49 μM. Tests in living HepG2 cells showed that the probe possesses high cell penetration to detect Cu^2+^ in cells sufficiently. While the authors provide a clear and detailed description of the chemosensor’s structural framework, it would be interesting to explore its application in other systems, aside from living cells. Moghadan et al. reported 2-[(9H-fluoren-2-ylmethylene)-amino]-phenol (**probe-21**), shown in Figure 7c, which is a fluorogenic Schiff base chemosensor designed for tracking Cu^2+^ [85]. **Probe-21** is weakly fluorescent because of C=N isomerization and ICT. When Cu^2+^ is added, both processes are suppressed, resulting in a turn-on response and a color change from pale yellow to colorless. The probe also proved highly selective towards Cu^2+^, with an LOD of 1.54 nM. While the relevant tests were performed and the results show a functional chemosensor, investigations on temperature stability, pH tolerance, and solvent compatibility are particularly important to determine its environmental robustness and assess its practical applicability.

### 4.3. Schiff Bases as Chemosensors for Fe^2+^ and Fe^3+^

Excessive Fe^2+^ in water can promote the growth of certain algae species, leading to algal blooms. These blooms can deplete oxygen levels, cause water turbidity, and create dead zones, affecting other aquatic organisms and ecosystems. In humans, high levels of Fe^2+^ in drinking water can result in iron overload, known as hemochromatosis. This can cause organ damage, particularly to the liver, heart, and pancreas. It can also lead to gastrointestinal distress, joint pain, and fatigue. Fe^3+^ is another cation that has been subject to intensive research. In the environment, Fe^3+^ can contribute to eutrophication in water bodies when it acts as a nutrient and promotes excessive algae and plant growth. This can lead to oxygen depletion, aquatic species imbalance, and deterioration of water quality. In humans, excessive exposure to Fe^3+^ can disrupt the iron balance in the human body. It can interfere with the absorption, utilization, and storage of iron, leading to iron overload or deficiency, which can have various negative health consequences. 

Schiff bases can selectively bind with Fe^2+^ ions and undergo a color change or fluorescence enhancement, allowing easy detection. The interaction between the Schiff base and Fe^2+^ can result in a coordination complex, altering the electronic and optical properties of the compound. This change is often observable via visual inspection or spectroscopic techniques. Schiff bases can also act as chemosensors for Fe^3+^ ions. Fe^3+^ has a high affinity for oxygen atoms, so Schiff bases with oxygen donor atoms, such as phenols or alcohols, can form stable complexes with Fe^3+^. The coordination of the metal ion can induce changes in the Schiff base’s electronic structure and spectroscopic properties, leading to a detectable signal such as color change or fluorescence quenching/enhancement. 

**Figure 8:**
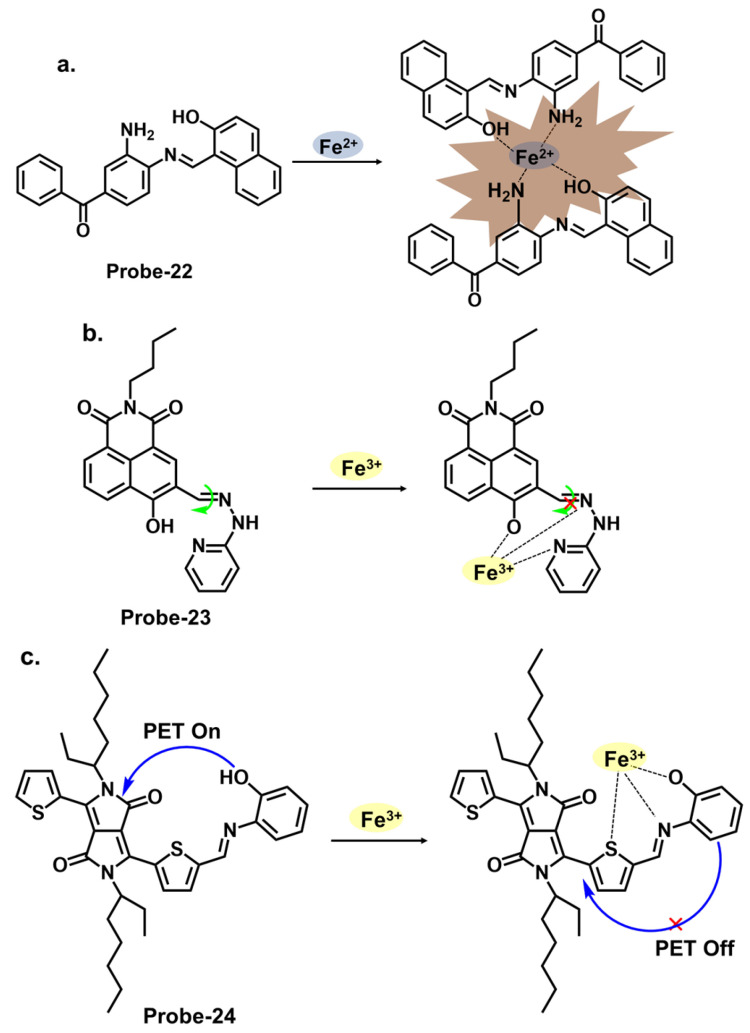
The structures and mechanisms of benzophenone-, pyridine-, and pyrrole-derived Schiff base chemosensors for Fe^2+^ and Fe^3+^; (**a**) **probe-22**, (**b**) **probe-23**, and (**c**) **probe-24**.

Figure 8a shows **probe-22** by Kouser et al., which is a benzophenone-derived Schiff base Fe^2+^ tracker prepared via a condensation reaction between 2-hydroxy-1-naphthaldehyde and 3,4-diamino benzophenone in methanolic solution [86]. The methodology section offers a detailed account of the synthesis process and characterization techniques utilized. Job’s plot, density Functional theory (DFT) calculations, and ^1^H NMR studies confirmed a 2:1 binding stoichiometry, while UV–vis titration estimated the detection limit at 0.0363 μM. Adding Fe^2+^ resulted in a calorimetric response, changing the color from light yellow to brown. In this case, the fluorescence enhancement resulted from the restriction of C=N isomerization processes and PET processes. When tested for application, **probe-22** detected Fe^2+^ in various water samples. While the sensitivity and selectivity of the chemosensor towards Fe^2+^ are thoroughly discussed, a more detailed exploration into the underlying reasons for the selectivity towards Fe^2+^ would be beneficial. The Schiff base receptor **probe-23**, shown in Figure 8b (naphthalic anhydride–(2-pyridine) hydrazone) by Hou et al., was synthesized by the condensation reaction of naphthalimide monaldehyde and 2-hydrazine pyridine to detect Fe^3+^ [87]. The reversible, water-soluble and high Stokes shift (100 nm) probe formed a 1:1 complex with Fe^3+^, suppressing C=N isomerism and causing a turn-on response. The sensitivity was quite high, with the LOD value estimated at 38.3 nM. **Probe-23** showed high anti-interference properties, while the working pH was in the range of 3.2 to 6.4. A detailed comparative study with existing fluorescence chemosensors was performed, which highlighted the unique properties of this sensor. As shown in Table 1, it is one of the few probes that was used in cancerous cells, as it is able to track Fe^3+^ in liver cancer cell bel-7420 with high cell permeability and low cytotoxicity. To further understand this probe, additional experiments, such as binding studies or computational modeling, could help elucidate the underlying sensor–Fe^3+^ interactions. Zhang et al. reported **probe-24**, shown in Figure 8c, which is a Schiff base Fe^3+^ calorimetric and fluorescent tracker based on a diketopyrrolopyrrole derivative synthesized by reacting the diketopyrrolopyrrole aldehyde with o-aminophenol [88]. The authors conducted a thorough sensitivity and selectivity assessment of the chemosensor towards a range of cations, providing an understanding of its capabilities. The probe is weakly fluorescent because of active PET processes. Adding Fe^3+^ changed the color from amaranth to rose pink. At the same time, the enhanced fluorescence resulted from the inhibition of PET and the onset of CHEF. Further analysis showed a binding constant value of 2.4 × 10^4^ M^−1^ and a very low LOD of 14.3 nM, with good anti-interference, reversibility, and response time. Furthermore, analysis of various spiked water samples showed recovery rates above 92%. 

**Figure 9:**
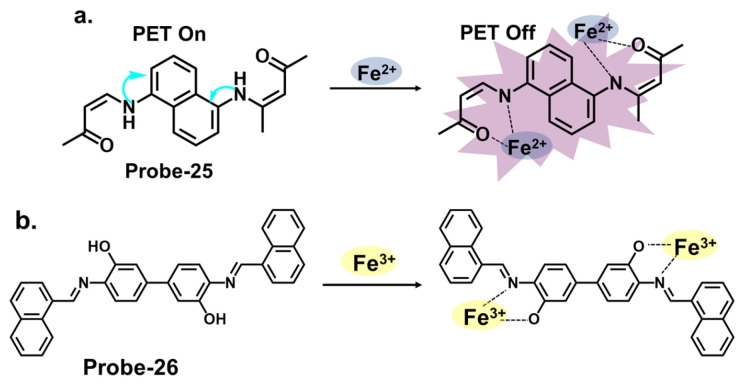
The structures and mechanisms of naphthalene- and dihydroxybenzidine-derived Schiff base chemosensors for Fe^2+^ and Fe^3+^; (**a**) **probe-25** and (**b**) **probe-26**.

Figure 9a shows **probe-25** by Selvan et al., which is a Schiff base sensor with bis-bidentate N, O sites synthesized from the condensation reaction between naphthalene-1,5-diamine and acetylacetone in ethanol [89]. The dual-signaling probe was used to track Fe^2+^ in a turn-on reaction due to suppression of the PET mechanism. It also detected Cu^2+^ via a charge transfer mechanism in a turn-off reaction. Emission titration experiments determined the binding ratios between **probe-25** and the ions to be 1:2, while the LOD for Fe^2+^ ions was estimated to be 0.5 μM. The probe showed high selectivity towards Fe^2+^, with negligible interference from other ions. While the study provides valuable insights into the design and mechanism, further validation with diverse real-world samples would provide a better understanding of its limitations. **Probe-26** by Zuo et al., shown in Figure 9b, is a turn-on Schiff base chemosensor prepared from 3,3′-dihydroxybenzidine and 1-naphthaldehyde in ethanol for the colorimetric and fluorescent detection of Fe^3+^ [90]. The authors provide a detailed overview of the synthesis methodology employed, highlighting the characterization techniques and spectroscopic analysis performed. **Probe-26** is weakly fluorescent because of active C=N isomerization and PET processes. The two processes are suppressed upon coordination with Fe^3+^, resulting in enhanced fluorescence and a color change from pale yellow to colorless. Job’s plot confirmed the binding ratio to be 1:2, while fluorescence titration spectra estimated the LOD to be as low as 0.178 μM, reflecting high sensitivity. For application, **probe-26** was successfully used in real water and food samples, with recovery rates above 98%. Furthermore, tests on paper strips also showed visible color changes in the presence of Fe^3+^, attributed to ligand-to-metal charge transfer (LMCT). 

**Figure 10:**
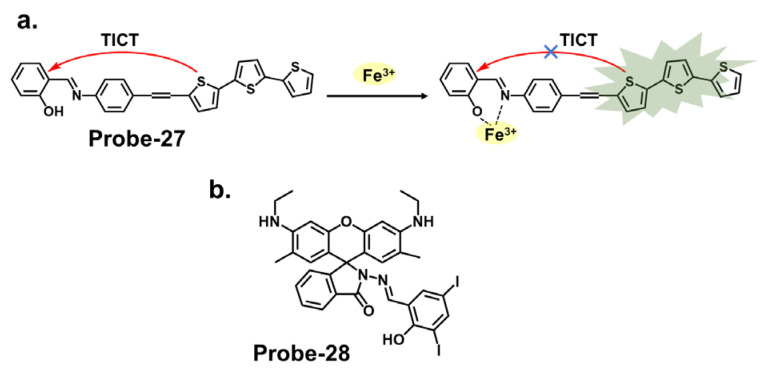
The structures (and mechanisms) of thiophene- and rhodamine-derived Schiff base chemosensors for Fe^3+^; (**a**) **probe-27** and (**b**) **probe-28**.

Thiophene derivatives can also be used in tandem with other functional groups to create cation-targeting probes, as shown in Figure 10a. Guo et al. reported **probe-27**, a dual-functional Schiff base chemosensor based on oligothiophene–phenylamine moieties synthesized to detect Al^3+^ and Fe^3+^ ions simultaneously [91]. **Probe-27** is weakly fluorescent because of twisted intramolecular charge transfer (TICT) processes. Adding Fe^3+^ resulted in a **probe-27**-Fe^2+^ complex and enhanced fluorescence due to suppression of the TICT process. The fluorogenic chemosensor detected Al^3+^ and Fe^3+^ with LOD values of 0.177 µM and 0.172 µM, respectively, with a color change from colorless to green. The Job’s plot confirmed 1:1 binding ratios for both ions, and the probe was proven operational in the pH range of 4.0 to 12. Furthermore, real water and food samples tests showed recovery rates above 97%. The findings presented in this study are interesting, and a deeper dive into the mechanistic features, such as rigidity or planarity, would further help explain the selectivity and the subsequent fluorescence activation. Rhodamine derivatives utilize the open and closed form to produce fluorescent changes which form the basis for detection. Figure 10b shows **probe-28** by Murugan et al., which is a Schiff base receptor prepared from rhodamine hydrazide and salicylaldehyde to detect Fe^3+^ and Cu^2+^ ions [92]. The turn-on reception mechanism results from the spirolactum ring cleavage, followed by ICT, resulting in enhanced fluorescence and a color change from colorless to red. The sensitivity was very high, with LOD values of 3.1 nM and 2 nM for Fe^3+^ and Cu^2+^ ions, respectively. Emission studies showed that **probe-28** possesses strong anti-interference properties, while Job’s plot analysis showed binding ratios of 1:1 for both metals. For application, **probe-28** detected Fe^3+^ in zebrafish embryos while showing negligible cytotoxicity. It would be worthwhile to study the solvent effects or other potential limitations or side reactions associated with the chemosensor’s fluorescence response to gain a better understanding of its applications.

### 4.4. Schiff Bases as Chemosensors for Zn^2+^

Zinc can enter water bodies through natural processes, such as weathering of rocks and soil erosion, as well as from human activities such as industrial discharges, mining, and agricultural runoff. Elevated zinc levels in water can be toxic to aquatic organisms, including fish, insects, and plants [108]. It can disrupt their physiological processes, impair growth and reproduction, and cause mortality. Zinc contamination can also affect the ecosystem balance and biodiversity in aquatic habitats [109]. Monitoring zinc levels can help understand its distribution and regulation within living organisms and its role in health and disease [110]. Chemosensors for zinc can provide valuable insights into zinc homeostasis and aid in developing therapies for zinc-related disorders.

**Figure 11:**
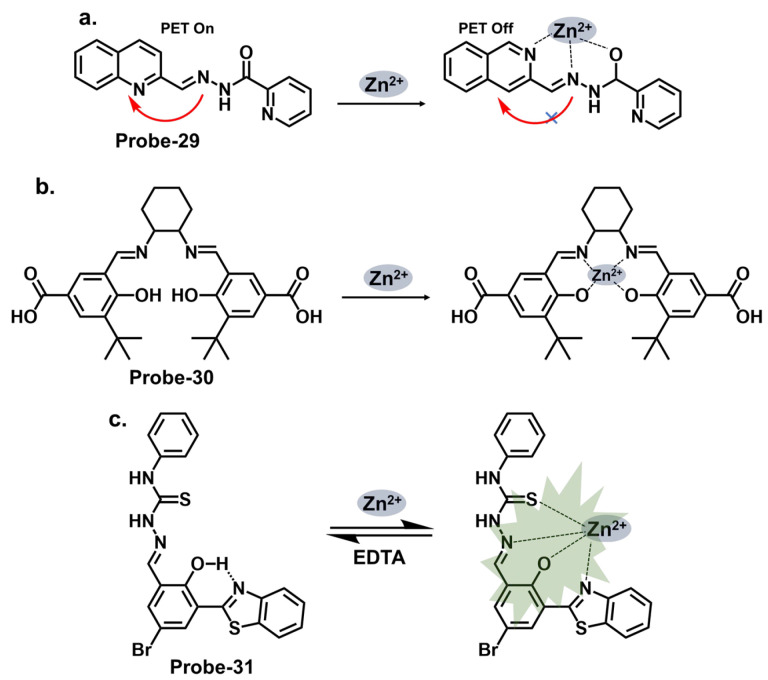
The structures and mechanisms of pyrrole-, hydroxybenzoic-acid-, and benzothiazole-derived Schiff base chemosensors for Zn^2+^; (**a**) **probe-29**, (**b**) **probe-30**, and (**c**) **probe-31**.

When pyrrole units are introduced into the Schiff base structure, they enhance the fluorescence response of the sensor. Pyrrole is a conjugated aromatic ring with a high electron density, allowing efficient π-electron delocalization [111]. This delocalization leads to enhanced fluorescence emission upon excitation. The functionalization of pyrrole units in the Schiff base chemosensors can be tailored to recognize specific analytes. For example, different substituents or functional groups can be introduced on the pyrrole ring to enable selective detection of metal ions, anions, or organic molecules [112]. The interaction between the analyte and the Schiff base sensor leads to changes in the electronic environment and fluorescence properties, providing a means for analyte detection and quantification. Wang et al. reported a condensation reaction between isonicotinohydrazide and ethyl 5-formyl-2,4-dimethyl-pyrrole-3-carboxylate in ethanol to produce a pyrrole-bearing Schiff base hydrazone chemosensor (**probe-29**) for the calorimetric and fluorescence tracking of Cu^2+^ and Zn^2+^, respectively (Figure 11a) [93]. CHEF was activated upon adding Zn^2+^, with a low LOD value of 0.18 μM. **Probe-29** also showed high cell penetration, as it was used to detect Zn^2+^ in U251 glioma cell lines. While the response to Zn^2+^ is presented with clarified calorimetric and fluorimetric changes, the range of analytes used to assess selectivity could be expanded further. He et al. reported the condensation of 1,2-cyclohexane diamine and 3-(tert-butyl)-5-formyl-4-hydroxybenzoic acid to prepare **probe-30** for the selective recognition of Zn^2+^ and pH changes (Figure 11b) [94]. The authors describe the synthesis and characterization of the Schiff base chemosensor in detail, including the reaction conditions and purification techniques employed. Spectroscopic analysis techniques such as UV–vis, fluorescence, and NMR are utilized for structural characterization. The blue luminescence observed was due to the suppression of PET processes. The LOD was determined to be 56 nM, indicating high sensitivity. For applications, **probe-30** proved functional in paper strips, zebrafish, and live cells. Benzothiazole has been used in several studies as a backbone for fluorescent chemosensors. The fluorescence response of benzothiazole Schiff base chemosensors relies on the coordination of binding events with target analytes. The imine or azomethine group in the Schiff base provides a binding site for various species through coordination chemistry, hydrogen bonding, or other interactions. When the target analyte binds to the Schiff base, it can induce changes in the benzothiazole moiety’s electronic structure or environment, leading to changes in the fluorescence properties. Wu et al. prepared a substituted Schiff base benzothiazole (**probe-31**) for the turn-on detection of Zn^2+^ (Figure 11c) [95]. The ratiometric sensor uses benzothiazole as a signaling moiety, while the Schiff base moiety is the receptor site. It is fluorogenic, as the color shifts from orange to green. The 1:1 interaction between the Zn^2+^ and the phenolic O inhibits the ESIPT process, leading to CHEF. Furthermore, it proved very sensitive, with the LOD estimated at 37.7 nM. While the spectral studies clearly show high selectivity towards Zn^2+^, reasons for non-selectivity towards other divalent ions such as Co^2+^ and Cu^2+^ could be investigated further.

**Figure 12:**
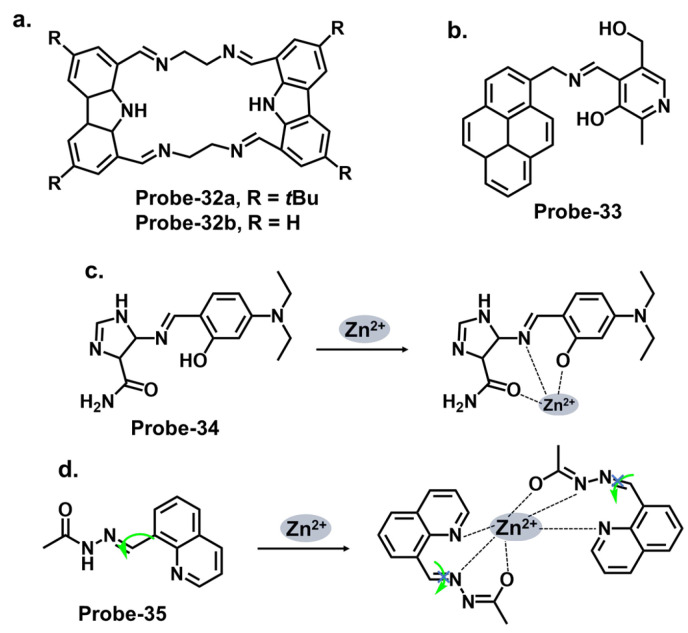
The structures (and mechanisms) of carbazole-, pyrenemethylamine-, imidazole-, and quinoline-derived Schiff base chemosensors for Zn^2+^; (**a**) **probes-32a**, **32b**, (**b**) **probe-33**, (**c**) **probe-34**, and (**d**) **probe-35**.

Carbazole macrocycles, shown in Figure 12a, are cyclic structures of multiple carbazole units which offer several advantages as chemosensors. The presence of multiple carbazole units within the macrocycle enhances the rigidity and stability of the structure, which is crucial for efficient fluorescent responses. The extended conjugation provided by the carbazole units allows for a redshift in the absorption and fluorescence spectra, making them suitable for various applications. They offer a promising platform for developing sensing systems with applications in various fields, including environmental monitoring, biological imaging, and chemical analysis. The combination of their inherent fluorescence properties and the versatility of Schiff bases provide ample opportunities for designing efficient and selective chemosensors. Malthus et al. used macrocycles derived from carbazole units to prepare highly selective Schiff base probes (**probes-32a** and **32b**) to detect Zn^2+^ [96]. The condensation reaction between ethylene diamine and carbazole dialdehyde produced two slightly different probes that relied on the suppression of PET and CHEF to produce a turn-on reaction in the presence of Zn^2+^. Overall, the probes had minimal interference, LODs in the nanomolar range, and strong binding, confirmed by ESI, ^1^H NMR, and DFT calculations. This study introduces new high-yielding and scalable reactions to form a new class of carbazole-based Schiff base macrocycles, whose applications could be explored further. Upadhyay et al. reported the condensation of 1-pyrenemethylamine with the vitamin B_6_ cofactor pyridoxal to create a three-in-one Schiff base probe (**probe-33**) for tracking Zn^2+^, hydrogen phosphate, and cysteine (Figure 12b) [97]. Again, the turn-on response originates from the suppression of the PET mechanism between the imine nitrogen and the electron-accepting pyrene fluorophore. The LOD values were sufficiently low (Table 1), determined to be 2.3 μM, 0.21 μM, and 0.16 μM for Zn^2+^, hydrogen phosphate, and cysteine, respectively. **Probe-33** was also functional, as it was successfully used to capture images of treated and untreated HeLa cells. An investigation into the response time across all three analytes will give a clearer assessment into its suitability for real-time monitoring and detection of analytes, which would have significant practical applications in areas such as environmental monitoring, healthcare, and food safety. 

Imidazole-based Schiff base compounds have also been explored as fluorescent chemosensors due to their unique characteristics and potential applications. Imidazole is a five-membered heterocyclic aromatic compound containing two nitrogen atoms (Figure 12c). By incorporating a Schiff base functionality, which includes an imine or azomethine group, imidazole Schiff bases can exhibit interesting fluorescence responses upon interaction with specific analytes. Yun et al. reported an imidazole derivative as a Schiff base probe (**probe-34**) for the turn-on-off detection of Zn^2+^, S^2−^, and Zn^2+/3+^ ions [98]. **Probe-34** was prepared from the condensation reaction between (4)-amino-4(5)-(aminocarbonyl)imidazole hydrochloride and 4-diethylamino salicylaldehyde in MeOH. PET between the moieties renders the probe weakly fluorescent. Adding Zn^2+^ disrupts the PET process, resulting in a turn-on fluorescence via CHEF. Job’s plot confirmed the 1:1 stoichiometry, while LOD values were estimated to be as low as 1.59 μM and 8.03 μM, respectively. Furthermore, the coordination proved reversible, as the addition of EDTA released the analyte. Probe-34 is one of very few sensors that can recognize four analytes. In fact, its reception to four analytes makes the probe vulnerable to false positives, so it would be worthwhile to investigate the underlying physical phenomena to gain understanding of the reported turn-on/-off mechanism.

Quinolines are versatile organic compounds that have been widely employed as fluorescent chemosensors. Their unique photophysical properties, including high quantum yields, large Stokes shifts, and tunable emission wavelengths, make them suitable candidates for sensing applications. By modifying the structure of quinolines, researchers can design chemosensors with specific recognition capabilities for various analytes. One common approach is introducing functional groups or receptors into the quinoline structure to form a Schiff base probe exhibiting selectivity towards a particular analyte. These functional groups can undergo specific interactions such as hydrogen bonding, coordination, or ion-pairing with the target analyte, leading to changes in the fluorescence properties of the quinoline. This fluorescence intensity, color, or lifetime change can be easily monitored and quantified, providing a reliable signal for analyte detection. Wu et al. reported a high-yield condensation reaction to synthesize a Schiff base chemosensor (**probe-35**) with a large Stokes shift (>200 nm) for the detection of Zn^2+^ in aqueous media in the micromolar range (Figure 12d) [99]. The probe itself is weakly fluorescent because of C=N isomerism. The introduction of Zn^2+^ inhibits the isomerization, as the probe coordinates with the ion in a 2:1 stoichiometry and an LOD on 89.3 nM. The probe was used to detect Zn^2+^ in HeLa cells with very low cytotoxicity for application. While spectral studies and computational models were performed to determine selectivity and mechanism, further investigations into the stability and photostability of the chemosensor under various environmental conditions, such as pH and temperature variations, would provide useful information for practical applications.

**Figure 13:**
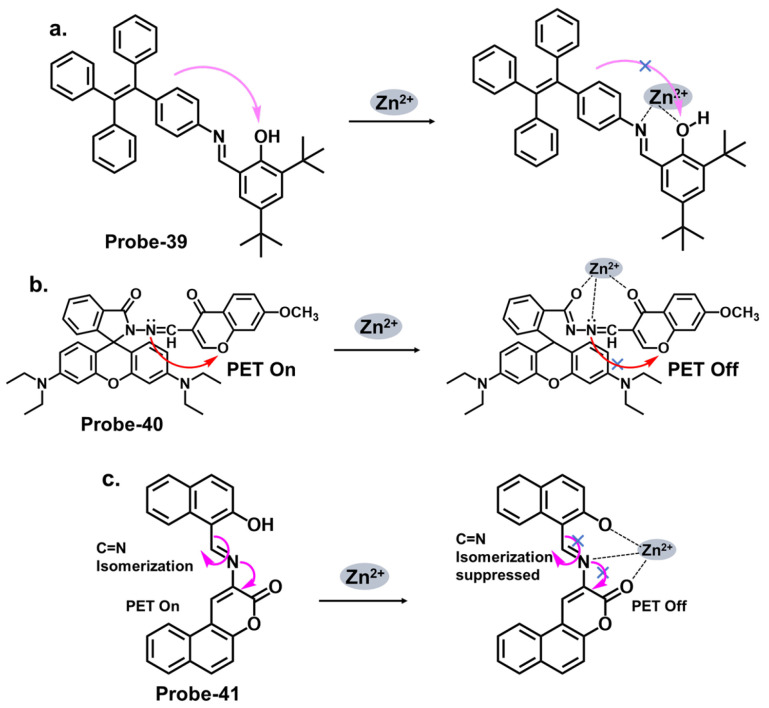
The structures and mechanisms of salicylaldehyde-, rhodamine-, and benzocoumarin-derived Schiff base chemosensors for Zn^2+^; (**a**) **probe-36**, (**b**) **probe-37**, and (**c**) **probe-38**.

Salicylaldehyde has been used as an anchoring group in various chemosensor designs [113]. Jia et al. reported **probe-36**, shown in Figure 13a, which is an AIE-active Schiff base receptor based on tetraphenylethylene-functioned salicylaldehyde for tracking Zn^2+^ [100]. The authors provide a clear and detailed description of the chemosensor’s structural framework, with the synthetic route and the characterization techniques adequately explained. The turn-on probe displayed reversible mechanofluorochromism, reflected by the emission color change from yellowish-green to orange-yellow after grinding. The enhanced fluorescence is due to the inhibition of the PET and the ESIPT processes once the Zn^2+^ ions were added. **Probe-36** also showed high sensitivity, reflected by an LOD value of 80.5 nM. Rhodamine Schiff bases are chemosensors derived from coupling rhodamine dyes with various aldehydes or ketones. Incorporating the Schiff base functionality introduces a new chromophore, leading to changes in the photophysical properties of the rhodamine dye. This alteration in the optical properties allows for detecting and quantifying specific analytes or ions in solution. The detection mechanism of rhodamine Schiff base chemosensors is based on molecular recognition. The imine linkage in the Schiff base structure can selectively interact with target analytes through various non-covalent interactions such as hydrogen bonding, coordination, or electrostatic interactions. These interactions lead to changes in the rhodamine dye’s absorption and/or emission spectra, providing a signal response that can be monitored spectroscopically. Liu et al. reported **probe-37** (7-methoxychromone-3-carbaldehyde-rhodamine B carbonyl hydrazone), shown in Figure 13b, for the turn-on detection of Zn^2+^ [101]. The probe incorporated the rhodamine and chromone moieties, which facilitated PET processes. The 1:1 coordination with Zn^2+^ inhibited the PET process, enhancing emission. The sensitivity was high, with the LOD value estimated at 0.34 μM. Its integration into paper strips produced a convenient and low-cost test kit for tracking Zn^2+^.

Coumarins exhibit fluorescence due to the presence of conjugated π-electron systems. When excited by light of a specific wavelength, they absorb energy and then emit light at a longer wavelength. The extent of fluorescence can be influenced by the surrounding environment, allowing for the development of fluorescent chemosensors. They are versatile building blocks for developing fluorescent chemosensors. Their inherent fluorescence, combined with appropriate modifications and receptor groups, enables the selective detection and quantification of various analytes, offering significant potential for applications in analytical chemistry and sensor technology. The fluorescence response of coumarin-based Schiff base chemosensors can be attributed to various mechanisms, including ICT, excited-state proton transfer, or heavy atom effect. These mechanisms can result in changes in the fluorescence intensity, emission wavelength, or lifetime of the coumarin moiety. Liu at al. synthesized a multi-analyte probe (**probe-38**) from 3-Amino-5,6-benzocoumarin and 2-hydroxy-1-naphthaldehyde for the detection of Zn^2+^, Cu^2+^, and S^2−^ (Figure 13c) [102]. The inherent intramolecular PET and C=N isomerization renders the probe poorly florescent. However, the addition of Zn^2+^ results in the suppression of these processes, leading to enhanced fluorescence. The binding was 1:1 for this structure, with an LOD value estimated at 3.6 μM. Like coumarins, pyrenes possess a conjugated π-electron system that imparts strong fluorescence properties. While the spectral studies were thoroughly performed to determine selectivity and interference, theoretical calculations could be explored to elucidate the underlying sensor–Zn^2+^/Cu^2+^/S^2−^ interactions.

**Figure 14:**
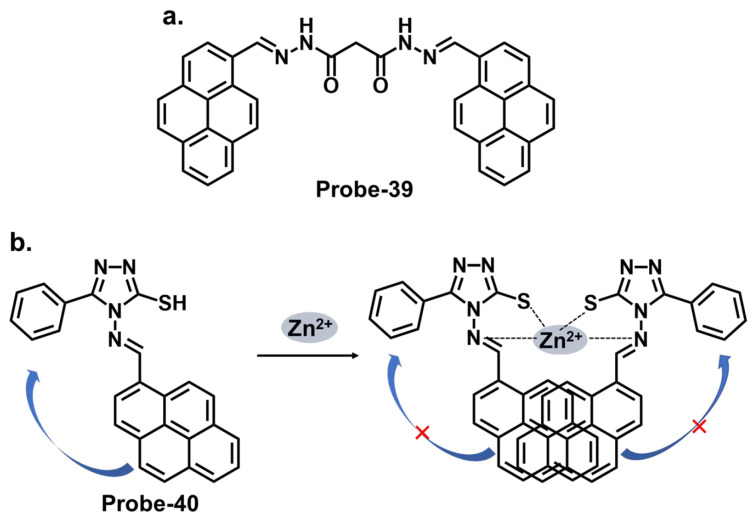
The structures (and mechanisms) of pyrene-derived Schiff base chemosensors for Zn^2+^; (**a**) **probe-39** and (**b**) **probe-40**.

Pyrene derivatives have also been extensively explored as chemosensors for various ions [86]. Rani et al. combined pyrene and malonohydrazide groups to develop a receptor (**probe-39**) for tracking Zn^2+^, as shown in Figure 14a [103]. Because Zn^2+^ and Cd^2+^ have similar spectral properties, this sensor was prepared specifically to distinguish between the two. The presence of donor (imine nitrogen) and acceptor groups (pyrene) ensured active PET processes, making the receptor weakly fluorescent. Adding Zn^2+^ disrupts the PET processes, as it attaches to the NH nitrogen in a 1:1 binding stoichiometry. **Probe-39** had low cytotoxicity in HeLa cells and exhibited enhanced fluorescence once Zn^2+^ was added. It would be interesting to extend the analysis to real-world samples, such as environmental water or other biological fluids, to fully assess the practical applications. Shellaiah et al. reported a one-pot reaction for synthesizing an AIEE-active pyrene-based Schiff base chemosensor (**probe-40**) for detecting Zn^2+^ and tyrosine in the solution [104]. Characterization data confirmed a successful synthesis. The probe, shown in Figure 14b, coordinated with Zn^2+^ in a 2:1 stoichiometry, suppressing PET processes and enhancing the fluorescence. The LOD was estimated to be 0.79 nM from fluorescence linear fittings. It also proved to be biocompatible and could track Zn^2+^ in B16F10 cell lines and zebrafish.

### 4.5. Schiff Bases as Chemosensors for Other Ions

**Figure 15:**
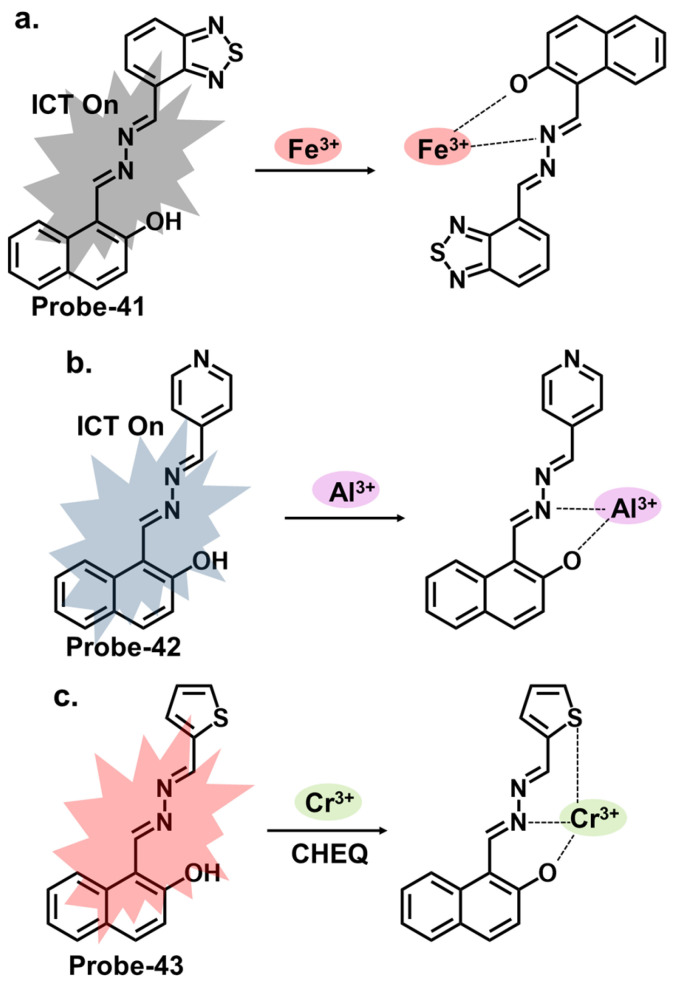
The structures and mechanisms of benzothiadiazole-, pyridine-, and thiophene-based Schiff base chemosensors for Fe^3+^, Al^3+^ and Cr^3+^, respectively; (**a**) **probe-41**, (**b**) **probe-42** and (**c**) **probe-43**.

Naphthyl hydrazones consist of a naphthyl group bonded to a hydrazine functional group (-NHNH_2_). Naphthyl hydrazones are utilized as fluorescent chemosensors for the detection of metal ions, anions, and biologically relevant species. The principles behind the use of these compounds in fluorescent chemosensors lie in their ability to undergo specific interactions or reactions with target analytes, leading to changes in their fluorescence properties. These changes can be measured and correlated with the analyte concentration or environmental conditions. Our group reported three naphthol-hydrazone-based Schiff base chemosensors for tracking Fe^3+^, Al^3+^, and Cr^3+^. **Probe-41**, shown in Figure 15a, contains a naphthol-hydrazine-based chemosensor skeleton covalently linked to the electron-withdrawing benzothiadiazole group [105]. The detailed experimental section enhances the reproducibility of this study and provides a valuable reference for researchers interested in developing similar structures. The probe is strongly fluorescent because of ICT. When Fe^3+^ is introduced, CHEQ is initiated, with a response time of 55 s and an LOD of 36 nM. **Probe-42**, shown in Figure 15b, is a naphthol hydrazone Schiff base receptor modified with pyridine to track Al^3+^ [106]. The extensive conjugation and active ICT render it strongly fluorescent. When Al^3+^ is present, ICT is suppressed, resulting in weaker emission. The LOD was estimated at 0.164 µM, while Job’s plot gave a 1:1 binding ratio. **Probe-43**, shown in Figure 15c, contains a naphthol hydrazone backbone attached to a thiophene ring [107]. The combined structure is an asymmetrical azine derivative, which was used to track Cr^3+^. The structure undergoes CHEQ in the presence of Cr^3+^, with no obvious interference from other ions. The affinity towards Cr^3+^ was explained by the HSAB theory; since Cr^3+^ is a hard acid, the ligand can be characterized as a hard Lewis base. The sensitivity was high, with the LOD estimated at 41 nM. For application, **probe-41** and **probe-43** were used to track Fe^3+^ and Cr^3+^ in PC3 cells, while **probe-42** was used to track Al^3+^ in HeLa cells.

## 5. Integration of Nanomaterials or Auxiliary Receptors

Furthermore, integrating nanomaterials or auxiliary receptors has shown promise in amplifying the detection efficiency of Schiff base chemosensors. Nanomaterials, such as metal nanoparticles, carbon nanotubes, or quantum dots, provide unique optical and electronic properties that enhance chemosensors’ sensitivity and signal amplification capabilities [114]. Researchers have improved selectivity, sensitivity, and stability by immobilizing the Schiff base chemosensors on the surface of these nanomaterials [115]. Additionally, incorporating auxiliary receptors, such as crown ethers, calixarenes, or cyclodextrins, has been explored to form supramolecular assemblies that selectively bind toxic cations. These auxiliary receptors act synergistically with the Schiff base chemosensor, enhancing sensing performance.

A particularly good property here is immobilization. Good immobilization is crucial in functional nanomaterial-based fluorescence chemosensors for several reasons:

1. Enhanced Stability: Immobilizing the functional nanomaterials ensures their stability and prevents their aggregation or leaching out. It helps maintain the nanomaterials’ structural integrity and fluorescence properties, leading to reliable and long-lasting sensor performance [116].
2. Improved Sensitivity: Immobilization can enhance the sensitivity of fluorescence chemosensors by providing a controlled environment for the nanomaterials. It minimizes background interference, reduces signal noise, and increases the signal-to-noise ratio, enabling the detection of trace amounts of target analytes with higher accuracy and lower LOD values [117].
3. Specificity and Selectivity: Functional nanomaterials can be modified with ligands or receptors to interact with specific analytes selectively. Immobilization allows for precisely positioning these recognition elements, promoting selective analyte binding and minimizing nonspecific interactions. This ensures high specificity and selectivity of the chemosensor towards the target analyte [118].
4. Easy Handling and Integration: Immobilization facilitates the handling and integration of functional nanomaterials into different sensor formats or platforms. It enables their incorporation into various devices such as lab-on-a-chip systems, wearable sensors, or surface-based arrays, enabling practical and convenient application in real-world settings [119].
5. Reusability: Immobilization can enable the regeneration and reuse of functional nanomaterials in sensing applications. For example, suppose the nanomaterials are immobilized on a solid support. In that case, they can be easily separated, regenerated, and reused after each sensing cycle, reducing the cost of sensor fabrication and operation [120].

### 5.1. Nanoparticle Materials-Based Chemosensors

Nanoparticles (NPs) have gained significant interest as building blocks for Schiff base chemosensors due to their unique physicochemical properties and high surface-to-volume ratios [121]. NPs provide a versatile platform for integrating Schiff base ligands, allowing for developing NPs-based Schiff base chemosensors with enhanced sensing capabilities. 

Various types of NPs can be employed in this context, including metallic NPs (such as gold, silver, or platinum), semiconductor NPs (such as quantum dots), magnetic NPs (such as iron oxide), and carbon-based NPs (such as graphene or carbon nanotubes) [122,123]. These NPs offer specific advantages depending on the target analyte and the desired sensing mechanism. The fabrication of NPs-based Schiff base chemosensors involves synthesizing or functionalizing NPs with Schiff base ligands [124]. The choice of ligands depends on the target analyte and the desired binding properties. The Schiff base ligands can be attached to the NPs through surface functionalization or coordination chemistry, providing a stable and selective sensing interface [125]. The interaction between the analyte and the Schiff base ligands on the NPs’ surface changes the physicochemical properties, including optical, electrical, magnetic, or catalytic properties [126]. For instance, in the case of metallic NPs, the presence of analytes can result in changes in the localized surface plasmon resonance (LSPR) or surface-enhanced Raman scattering (SERS) signals, which can be detected and quantified. 

The sensitivity and selectivity of NPs-based Schiff base chemosensors can be further enhanced by optimizing the size, shape, and surface chemistry of the NPs and the design of the Schiff base ligands [127]. Integrating NPs with specific recognition elements can enable the detection of various analytes, including heavy metal ions, organic compounds, biological molecules, and gases. The advantages of NP-based Schiff bases include their high sensitivity, rapid response, and compatibility with various detection techniques. Additionally, the tunability of NPs’ properties and the versatility of Schiff base ligands offer opportunities to develop customizable and selective sensing platforms [128]. However, challenges associated with these chemosensors include potential aggregation or instability issues, optimization of ligand–NP interactions, and potential interference from complex sample matrices.

#### 5.1.1. Nanoparticles-Based Chemosensors for Hg^2+^

**Figure 16:**
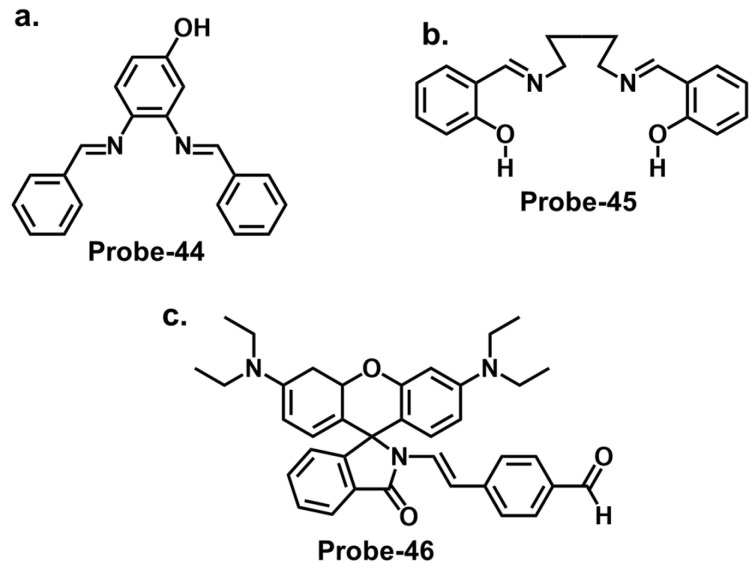
The Schiff base structures attached to the NPs for tracking Hg^2+^; (**a**) **probe-44**, (**b**) **probe-45**, and (**c**) **probe-46**.

A few representative nanoparticles-based chemosensors for Hg^2+^ have been reported in recent years. Gold nanoparticles, in particular, have exceptional optical properties and have been subject to extensive research regarding their modulation [129]. For instance, Amourizi et al. prepared a colorimetric sensor (**probe-44**) for the detection of Hg^2+^ ions utilizing surface-modified gold nanoparticles (GNPs) [130]. The probe, shown in Figure 16a, is functionalized with a Schiff base ligand. It can track Hg^2+^ because of the coordination between Hg^2+^ ions and azomethine nitrogen and the carbonyl oxygen of the ligand. The affinity of Hg^2+^ ions towards the bidentate Schiff base on the GNPs results from their self-aggregation, as the GNPs carry a negative charge. Additionally, it has a signal transduction pathway that leans on multivalent interactions between the recognition moiety and the GNPs. Combining these factors leads to a relatively low LOD (Table 2), making the probe suitable for tracking Hg^2+^ in drinking water. The gold nanoparticles were effective as they have been reported to enhance permeability, prove biocompatibility, and increase retention. Whether the probe can retain its sensitivity over time and under various environmental conditions is crucial for its practical applications. Another example of how surface modification of nanoparticles improves performance is shown in Figure 16b, which illustrates **probe-45** by Minhaz et al. [114]. They stabilized the Schiff base ligand on silver nanoparticles for detecting Hg^2+^ in the range of 0.01 μM to 50 μM. Finding ways to enhance the signal-to-noise ratio can further improve the detection limit and accuracy of the probe. The probe showed anti-interference properties in tap water and antibacterial and anticancer properties in the tested strains. Figure 16c shows **probe-46** by Mahajan et al., a rhodamine-based Schiff base that was used in a re-precipitation procedure to synthesize organic nanoparticles [131]. The Hg^2+^ tracker had a turn-on response from CHEF, with an LOD of 8.6 nM. As shown in Table 2, this LOD value compares well with other reported structures. The probe was successfully tested for functionality in various water samples and A375 living cells. Further investigations can be performed to improve the bioconjugation of this probe.

#### 5.1.2. Nanoparticles-Based Chemosensors for Cu^2+^

**Figure 17:**
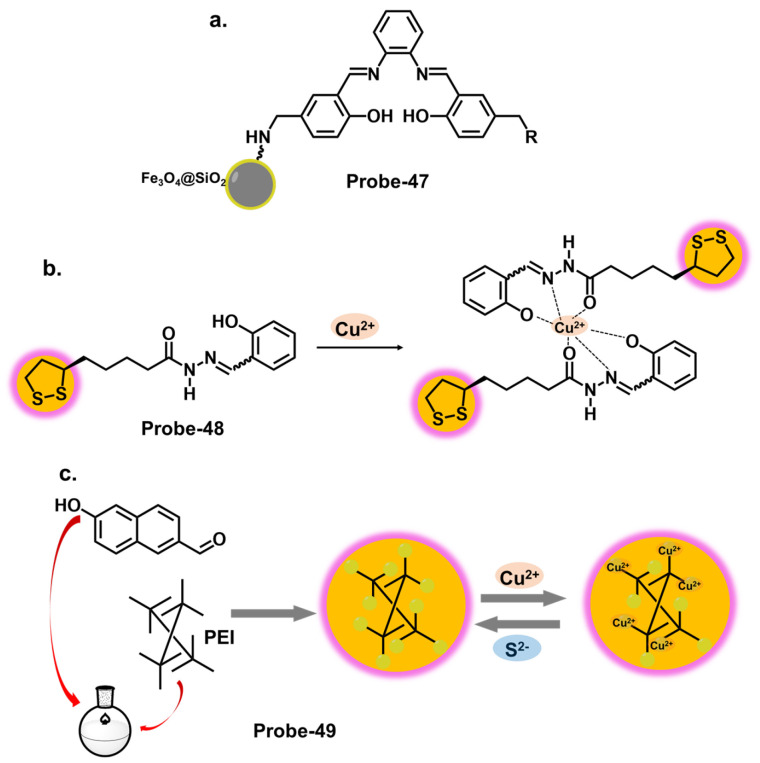
The Schiff base structures attached to the NPs for tracking Cu^2+^; (**a**) **probe-47**, (**b**) **probe-48**, and (**c**) **probe-49**.

Chemosensors can be grafted to nanoparticles to improve the efficiency of monitoring copper concentrations in wastewater, effluents, and other industrial waste streams. Silica nanoparticles have a large surface area, which allows for more interaction sites with analytes. This increases sensitivity of the sensor. Figure 17a illustrates **probe-47** by Zhu et al., which is a fluorimetric nanosensor based on the bis-Schiff base fluorophore for tracking Cu^2+^ [117]. The fluorophore was immobilized on silica-coated Fe_3_O_4_ nanoparticles via covalent bonding to create a recyclable matrix that can effectively enhance detection. The result was a highly functional and sensitive probe with an LOD value of 5.4 nM. From Table 2, this LOD is sufficiently low compared to other comparable structures. This probe can still be modified further to enhance its multiplexing capabilities by incorporating different sensing elements or using multimodal nanomaterials.

Gold is another class of nanoparticles which can be easily synthesized with controlled size and surface properties, making them attractive platforms. The inherent binding-induced aggregation or disassembly often results in a measurable signal. A study by Conkova et al. reports a multiprobe sensor based on a Schiff base ligand (**probe-48**) for the detection of Cu^2+^, Ni^2+^, and Fe^2+^ in the micromolar range, as illustrated in Figure 17b [132]. The fluorophore was chemisorbed on monodispersed gold nanoparticles to create a probe with high chemical stability, surface-to-volume ratio, and distinctive optoelectronic properties. This unique structure allowed for fluorogenic detection, with quick response and low LOD (1.4–11.2 nM). Hyperbranched polyethylenimine (hPEI) is a cationic polymer with abundant amine groups used to detect pH, metal ions, and biological enzymes [148,149,150]. It is easily modifiable and has been used to develop nanoprobes with detection ability via the Schiff base reaction. Yang et al. used a Schiff base reaction between hPEI and 6-hydroxy-2-naphthaldehyde to prepare highly functional fluorescent polymer nanoprobes (**probe-49**, Figure 17c) for the detection of Cu^2+^ and S^2−^ [133]. The probe could detect Cu^2+^ in 30 s, with an LOD of 243 nM. It was further used for bioimaging purposes in living cells and exhibited high cell penetration. The fluorescence quenching resulted from electron transfer between the amine group and Cu^2+^.

#### 5.1.3. Nanoparticles-Based Chemosensors for Other Ions

**Figure 18:**
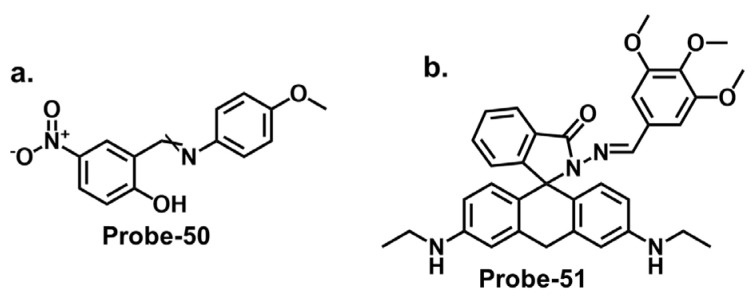
The Schiff base structures attached to the NPs for tracking Fe^3+^, Cr^2+^,Cd^2+^, and Pb^2+^; (**a**) **probe-50** and (**b**) **probe-51**.

NPs, particularly transition metal sulfide NPs, have been used to amplify the qualities of Schiff base chemosensors for sensing many other metal ions, as they have tailored surface properties, narrow size distribution, and wide band gap [151]. Ayodhya et al. reported a chemosensor (**probe-50**) comprised of zinc sulfide nanoparticles and a Schiff base capping agent, 2-[(4-methoxy-phenylamino)-methyl]-4-nitrophenol, as shown in Figure 18a [134]. The fluorometric multianalyte probe was used for detecting Fe^3+^, Cr^2+^, and Cd^2+^ in a turn-off response. The NPs complemented the Schiff base, resulting in the fast electron transfer between the surface functional imine group and metal ions. 

As previously mentioned, rhodamine dyes are used in fluorescent chemosensors because of their ability to exist in equilibrium between two forms. This allows the open and closed forms to emit optical changes that allow detection. When used in synergy with gold nanoparticles, it is conceivable that this signal could be amplified. Srisukjaroen et al. reported a calorimetric Schiff base Pb^2+^ tracker (**probe-51**), synthesized from the reaction between rhodamine 6G hydrazide and 3,4,5-trimethoxybenzaldehyde, shown in Figure 18 [135]. The probe alone showed a color change from colorless to pink within 60 s in the presence of Pb^2+^. When the probe was functionalized with (3-aminopropyl) triethoxysilane and gold nanoparticles, the color intensity increased 9-fold, with an LOD of 0.12 nM. For application, the probe was grafted onto paper strips and used to analyze Pb^2+^ in meat samples, with results consistent with those obtained from inductively coupled plasma-mass spectrometry method. While these results are impressive, investigating its stability over time and under different environmental conditions would provide a deeper understanding of its limitations. Its performance should be reproducible across multiple experiments.

Quantum dots are nanoscale semiconductor particles with unique optical and electronic properties. They are typically composed of inorganic materials, such as cadmium selenide (CdSe), lead sulfide (PbS), or indium phosphide (InP). One of the defining characteristics of quantum dots is their size-dependent bandgap. This means that the energy gap between the highest occupied molecular orbital (HOMO) and the lowest unoccupied molecular orbital (LUMO), which determines the electronic and optical properties of the material, changes as the size of the QDs changes. Due to their small size, QDs exhibit quantum mechanical behavior, leading to unique optical properties. They have a broad absorption spectrum, effectively capturing photons across various wavelengths. Additionally, they have a narrow and tunable emission spectrum, with the emission wavelength determined by the size of the QDs. QDs also possess a high quantum yield, efficiently converting absorbed photons into emitted photons, resulting in bright fluorescence. The emission color of QDs can be tuned by controlling their size, thus producing a wide range of colors. Integrating quantum dots with Schiff base chemosensors can synergistically improve the sensor system’s efficiency, sensitivity, selectivity, stability, and multiplexing capabilities, making it a promising approach for advanced sensing applications. Fan et al. prepared a luminescent fluorescent probe for the detection of picric acid by immobilizing a Schiff base chromophore on graphene quantum dots [152]. The probe had a quantum yield seven times higher than the Schiff base. The naked eye turn-off detection, which had an LOD value of 36.4 nM, resulted from energy and charge transfer between the electron-donating groups in the probe and the picric acid.

### 5.2. Nanoporous Materials-Based Chemosensors

Nanoporous materials have attracted significant attention in developing chemosensors due to their high surface area, large pore volume, and tunable pore size [153]. These materials provide a unique platform for the immobilization of Schiff base ligands, resulting in improved efficiency of the chemosensors. These nanoporous materials can act as a reservoir, entrapping and concentrating the analytes. Materials used in nanoporous materials-based Schiff base chemosensors include metal-organic frameworks (MOFs), covalent-organic frameworks (COFs), zeolites, mesoporous silica, carbon nanotubes, and porous polymers [154]. These materials offer a wide range of properties and can be tailored for specific applications [155]. Fabricating nanoporous materials-based Schiff base chemosensors involves synthesizing or selecting a suitable nanoporous material and subsequent functionalization with Schiff base ligands.

Schiff base ligands can be immobilized through covalent bonding, coordination with metal centers within the nanoporous material, or physical encapsulation [156]. The nanoporous structure of these materials provides many active sites for analyte adsorption and interaction with the Schiff base ligands. The binding between the target analyte and the Schiff base ligands can result in changes in the chemosensor’s optical, electrical, or mechanical properties, allowing for the detection and quantification of the analyte. The sensitivity and selectivity of such chemosensors can be further enhanced by selectively tuning the properties of the nanoporous material and the Schiff base ligands [157]. The ligands’ pore size, surface chemistry, and functional groups can be optimized to optimize the recognition and binding affinity towards the target analyte [158]. The advantages of nanoporous materials-based Schiff base chemosensors include high sensitivity, large surface area, and excellent control over pore size and surface functionalization. Furthermore, these chemosensors can be easily integrated into different detection platforms, including optical, electrochemical, and mass-based sensors. However, challenges associated with such chemosensors include the stability of the nanoporous structure under harsh conditions, the potential for nonspecific interactions, and the optimization of ligand immobilization techniques.

#### 5.2.1. Nanoporous-Based Chemosensors for Hg^2+^

**Figure 19:**
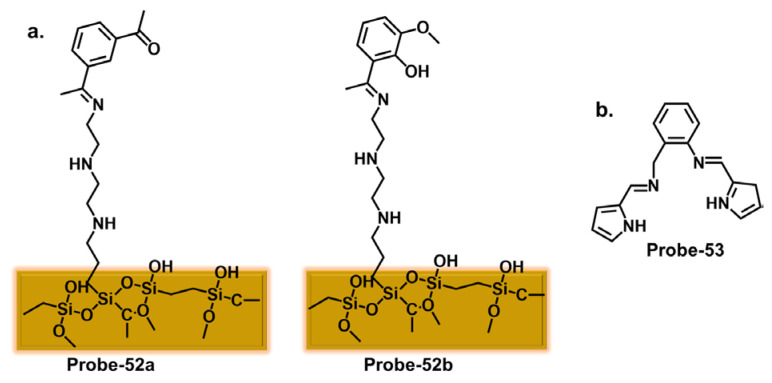
The Schiff base structures attached to the nanoporous materials for tracking Hg^2+^; (**a**) **probes-52a**, **52b**, and (**b**) **probe-53**.

Highly porous periodic mesoporous organosilica provides a large surface area that anaytes can interact with to induce optical and fluorescence changes. The sensing mechanism can involve modulation of charge transfer, energy transfer, or conformational changes. Figure 19a shows two Schiff base ligands functionalized with 2,6-diacetyl pyridine and o-vanillin, synthesized by Kascmarek et al. [136]. The two ligands were immobilized on periodic mesoporous organosilica to create functional probes (**probe-52a** and **52b**) for the turn-on detection of Hg^2+^. The nanoparticles were unique because the particle size could be altered by adjusting the synthesis procedure, thus potentially allowing it to be tailor-made for different Schiff bases. Another highly useful but different class of nanoporous materials is graphite. Modified graphite tends to increase the stability of the chemosensor, limiting degradation and aggregation. An example is a study by Selvan et al., who modified graphite electrodes with asymmetrical N4 tetradentate Schiff base ligand *N*,*N*′-bis(pyrrole-2-ylmethylene)-2-amino benzylamine for the simultaneous detection and recovery of Pb^2+^ and Hg^2+^ ions in sea and lake water (**probe-53**) [137]. The sensor, shown in Figure 19b, showed high stability and sensitivity, with LOD values of 1.1 nM for Pb^2+^ and 0.36 nM for Hg^2+^. Since graphite is a decent stabilizer, a more detailed investigation into the probe’s longevity will provide a good reference point for other researchers.

#### 5.2.2. Nanoporous-Based Chemosensors for Cu^2+^

**Figure 20:**
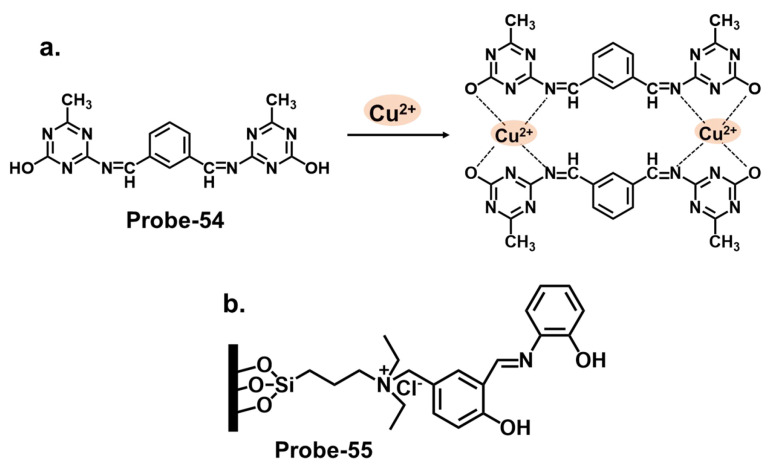
The Schiff base structures attached to the NPs for tracking Cu^2+^; (**a**) **probe-54** and (**b**) **probe-55**.

Cu^2+^ is a commonly targeted analyte in chemosensors due to its wide range of applications and potential impact on health and the environment. Because of this, research on specific ligands that selectively complex with Cu^2+^ is always evolving. Highly porous materials provide accessible mesopores, which facilitate complexation. Parsaee et al. immobilized a triazine-based Schiff base sensor on glass slides for tracking Cu^2+^ in aqueous media (**probe-54**) [138]. The calorimetric nano-sized sensor, shown in Figure 20a, was prepared via a sonochemical sol–gel process and could detect Cu^2+^ in the range OF 85.4 nM to 10 μM, with a relatively low LOD of 15.3 nM. In contrast, Zhang et al. prepared fluorescence **probe-55** based on quaternized salicylaldehyde Schiff base-modified mesoporous silica for the tracking and recovering of Cu^2+^ (Figure 20b) [139]. The highly selective and fluorogenic nanosensor facilitated the detection of Cu^2+^ via a combination of dynamic and static quenching, with an LOD of 0.37 μM. The quaternized linkage improved the dispersion and wettability, thus allowing the Schiff base moiety to determine the analyte’s concentration in water accurately.

#### 5.2.3. Nanoporous-Based Chemosensors for Fe^3+^

**Figure 21:**
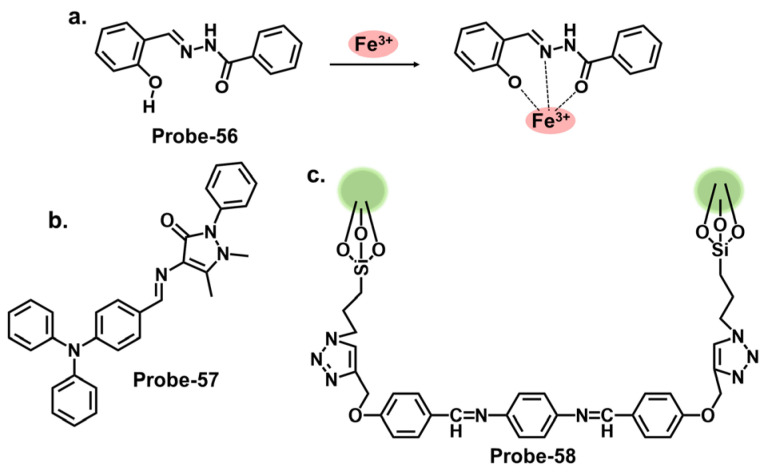
The Schiff base structures attached to the nanoporous materials for tracking Fe^3+^; (**a**) **probe-56**, (**b**) **probe-57**, and (**c**) **probe-58**.

The strength of the covalent bond inherent in COFs results in a material with a rigid, low density and highly stable structure with an established, permanent porosity. This forms a stable framework that can be functionalized with functional ligands. Chen et al. reported a hydrazone-linked COFs incorporating O, N, and O′-chelating sites for Fe^3+^ detection in water (**probe-56**) [140]. The luminescent Schiff base sensor, shown in Figure 21a, was prepared by a condensation reaction between 2,5-dimethoxyterephthal-aldehyde and benzene-1,3,5-tricarbohydrazide. When tested, it had a specific coordination reaction with Fe^2+^, resulting in a turn-off mechanism. **Probe-56** also showed strong luminescence in solid and aqueous solutions, making it suitable for Fe^3+^ detection in aqueous solutions.

Under certain conditions, Schiff base chemosensors can be susceptible to hydrolysis. This occurs when water molecules cleave the imine bond in the Schiff base, forming the corresponding amine and aldehyde or ketone. Silica cross-linked micellar NPs can deter this phenomenon. In a different but also equally interesting study, Gai et al. encapsulated a Schiff base derivative, (4E)-4-(4-(diphenylamino) benzylideneamino)-1,2-dihydro-1,5-dimethyl-2-phenylpyrazol-3-one, in silica cross-linked micellar nanoparticles to detect Fe^3+^ in aqueous medium and living cells (**probe-57**) [141]. The turn-off chemosensor, shown in Figure 21b, was water-compatible and highly sensitive, with a competitive LOD value of 0.21 µM. Figure 21c illustrates **probe-58** synthesized by Singh et al., who immobilized Schiff-base-derived bis1,2,3-triazolyl-γ-propyltriethoxysilanes on silica nanospheres for the dual detection of Fe^3+^ and Cu^2+^ [118]. Schiff-base-linked organosilanes, known to be structurally flexible, possess lone pairs on the nitrogen and oxygen atoms. Once immobilized of the silica nanospheres, better performance is observed as the organic–inorganic hybrid nanoparticles enhance electron transfer from the donor groups to the metal cations. This is reflected in the relatively low LOD values of 0.44 µM and 0.87 µM for Fe^3+^ and Cu^2+^, respectively.

#### 5.2.4. Nanoporous-Based Chemosensors for Other Ions

**Figure 22:**
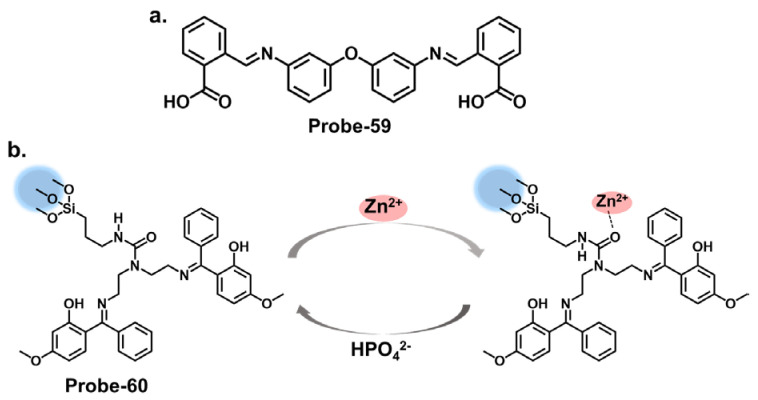
The Schiff base structures attached to the nanoporous materials for tracking Al^3+^ and Zn^2+^; (**a**) **probe-57** and (**b**) **probe-58**.

Some Schiff bases can be used directly to synthesize some nanoparticles. From Figure 22a, Mahmood et al. prepared a copper MOF with a carbon paste electrode and a nano-Schiff base linker for the potentiometric determination of the Al^3+^ ion in polluted water (**probe-59**) [142]. The electrode was successfully used with high precision (RSD% = 0.82–1.98) and accuracy (recovery% = 98.0–101.2). It was also proved to be fast (6 s) and very sensitive, as reflected by the low LOD of 33.1 nM. This improved efficiency can be attributed to the unique porous structure and high specific surface area, which allows high-precision detection of Al^3+^ in water samples. Silica spheres have been reported to possess permeability, low toxicity, and water dispersion properties. Their surfaces can be easily modified to incorporate various functional groups. Kaur et al. reported a reusable multifunctional inorganic–organic hybrid silica nanoprobe functionalized with a silatranyl derivative of Schiff base (**probe-60**), shown in Figure 22b [143]. The fluorogenic probe, grafted on nanosilica to improve stability in aqueous solutions, could identify, quantify, and recover Zn^2+^. Here, the ureido group facilitated the interaction with Zn^2+^, resulting in a turn-on response.

### 5.3. Metal Nanoclusters-Based Chemosensors

Metal nanoclusters have emerged as promising materials for developing chemosensors due to their unique optical and electronic properties [159,160,161]. Schiff bases can exhibit enhanced sensing capabilities towards various analytes when combined with metal nanoclusters. The metal nanoclusters act as signal transducers in these chemosensors [162]. They can undergo significant changes in their optical and electronic properties upon interaction with specific analytes. The fabrication of metal nanocluster-based Schiff base chemosensors involves the synthesis of well-defined metal nanoclusters and their functionalization with Schiff base ligands. The choice of metal nanoclusters and Schiff base ligands depends on the specific analyte to be detected. For example, silver or gold nanoclusters are often used due to their excellent fluorescence properties and high stability [163]. The interaction between the metal nanoclusters and analyte molecules can occur through various mechanisms, such as coordination or electrostatic interactions. This interaction leads to changes in the optical properties of the nanoclusters, including fluorescence intensity, emission wavelength, or lifetime. These changes can be measured using spectroscopic techniques, allowing for the detection and quantification of the analyte.

**Figure 23:**
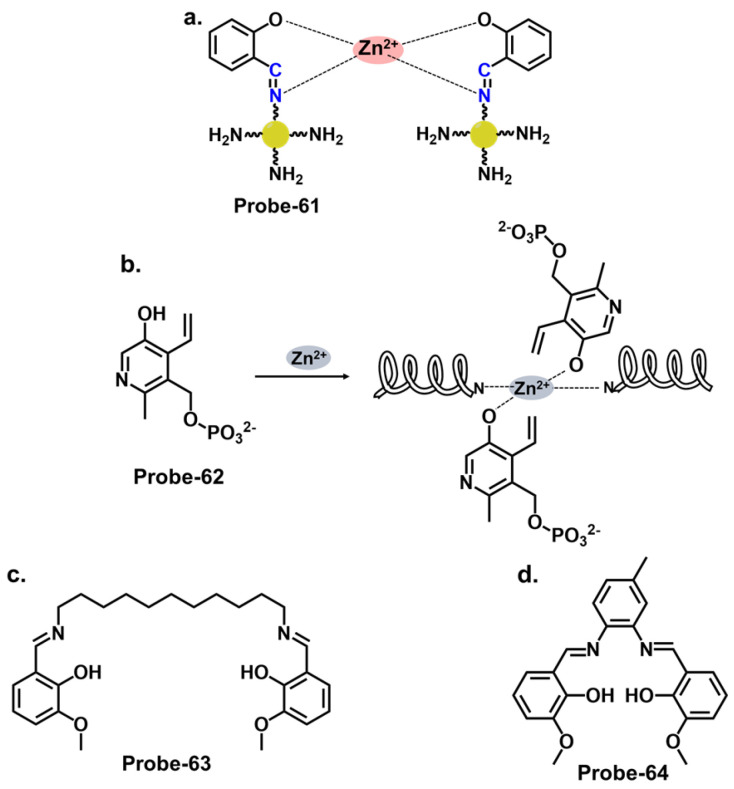
The Schiff base structures attached to metal nanoclusters for tracking Zn^2+^, Fe^2+^, and Co^2+^; (**a**) **probe-61**, (**b**) **probe-62**, (**c**) **probe-63**, and (**d**) **probe-64**.

The combination of Schiff base chemosensors and gold nanoclusters can provide signal amplification in the sensing process. The presence of an analyte can induce aggregation or disaggregation of the gold nanoclusters, leading to significant changes in the fluorescence signal. This amplification effect enhances the detection sensitivity and enables the detection of analytes at lower concentrations. Liu et al. prepared a Schiff base fluorescence sensor, **probe-61**, based on bovine-serum-albumin-protected gold nanoclusters to detect Zn^2+^ and salicylaldehyde, as shown in Figure 23a [144]. The coordination between Schiff base ligands and Zn^2+^ resulted in reduced charge transfer efficiency and inhibition of C=N isomerization, leading to a blue shift. Furthermore, the detection could be observed with the naked eye in the linear range of 0.1 μM to 100 μM, with an LOD of 29.28 nM (Table 2). In another study, Bothra et al. reported a red fluorescence nanoprobe of cocooned gold nanoclusters (**probe-62**) for detecting Zn^2+^ and vitamin B6 cofactor, pyridoxal-5′-phosphate (PLP) [145]. The Schiff base, shown in Figure 23b, is a product of the reaction between the -CHO of the PLP and the free -NH_2_ in the lysozyme. When Zn^2+^ is added, the result is a turn-on fluorescence reaction and a color change to yellow. It was also proved to be highly sensitive, as shown by the low LOD of 39.2 nM.

Cd–Ln nanoclusters possess both luminescent and magnetic properties. The luminescent property arises from the doped lanthanide ions, while the magnetic property is attributed to Cd and lanthanide elements. This dual functionality allows for the development of multifunctional sensors. Schiff base chemosensors can modify the luminescence properties of Cd–Ln nanoclusters. This alteration can lead to enhanced emission intensity, longer luminescence lifetime, or changes in emission wavelength upon interaction with the target analyte. Wang et al. synthesized Cd–Ln clusters from Schiff base ligands bearing a long methylene group skeleton for monitoring Fe^2+^ and Zn^2+^ (**probe-63**) [146]. The clusters, shown in Figure 23c, are unique because they are unusually large d–f Schiff base nanoclusters, favoring the encapsulation of Fe^2+^ and Zn^2+^. Liu et al. used a Schiff base ligand (**probe-64**) from the reaction between 2-hydroxy-3-methoxybenzaldehyde and 4-methylbenzene-1,2-diamine to synthesize a lanthanide nanocluster complex [Nd_3_L_3_(OAc)_3_] with a “triple-decker” structure for tracking Co^2+^, as shown in Figure 23d [147]. The result was a unique chiral lanthanide complex with NIR luminescence and a very low LOD of 0.97 μM.

## 6. Concluding Remarks

In this review, we have explored a range of strategies to improve the selectivity and sensitivity of Schiff base fluorescent chemosensors for detecting toxic and heavy metal ions. The different mechanisms and the underlying photophysical phenomena behind them influences chemosensor design. For instance, PET relies on electron transfer, AIE-gen is based on the restriction of intramolecular motion and prevention of non-radiative decay pathways, while ESIPT is a photophysical process that occurs in certain fluorophores such as coumarin derivatives, where light-induced proton transfer leads to significant changes in fluorescence properties. By critically evaluating the literature of several Schiff base chemosensors, we have identified several key approaches, including functional group variations, structural modifications, and the integration of nanomaterials or auxiliary receptors. Functional group variations have been highlighted as an effective strategy to enhance selectivity and sensitivity of Schiff base sensors. The modulation of binding affinity towards target cations can be accomplished through meticulous design and incorporation of specific functional groups into the Schiff base structure. The inclusion of electron-donating or electron-withdrawing groups, along with chelating moieties, serves to greatly enhance the chemosensor’s performance. Rational design of the binding pocket to optimize interactions with the target cation is another approach. This can involve introducing specific binding sites, modifying the cavity size, or incorporating steric hindrance to exclude interfering cations.

As a practical example, in rhodamine-based Schiff base chemosensors that target Hg^2+^, simply incorporating thiol (-SH) or amine (-NH_2_) functionalities into the rhodamine dye structure may not be enough. Researchers can enhance the spacing and functionalization of the linker between the rhodamine dye and the receptor unit. The linker can be designed to provide an ideal distance and arrangement for efficient Hg^2+^ binding and signal output. The structure could also be modified to stabilize the spirolactam form of the rhodamine dye, which is non-fluorescent. In the presence of Hg^2+^, the spirolactam ring opens, resulting in a highly fluorescent merocyanine form. Enhancing the stability of the spirolactam form will improve the selectivity and sensitivity of the chemosensor. The probe could also be modified to increase its rigidity, as this can enhance selectivity by reducing non-specific interactions with other cations. Furthermore, novel selectivity filters could be introduced within the receptor unit to discriminate against other metal ions that may interfere with Hg^2+^ detection. Researchers can design these filters to favor Hg^2+^ binding over other competing ions, thereby enhancing the selectivity and accuracy of the chemosensor.

Since these modifications depend on the chemosensor design and experimental conditions, a combination of multiple strategies may also be necessary to achieve the desired improvements. Stabilizing Schiff base compounds on nanomaterials has also been shown to improve efficiency as they can be excellent signal amplifiers, enhancing fluorescence emission upon cation binding. Surface functionalization of nanomaterials with Schiff base structures can impart selectivity towards specific cations. Future research should consider the exploration of novel nanomaterials to incorporate these Schiff base compounds. Improving the biocompatibility of the Schiff base chemosensors is also important for their applications in biological and medical fields. Nanomaterial modifications can and should focus on reducing potential cytotoxicity and enhancing bioconjugation strategies. Integrating these strategies will ultimately pave the way for the creation of highly efficient chemosensors, enabling accurate detection and monitoring of toxic cations across various applications. In summary, future research should consider the following steps to improve performance of the Schiff base chemosensors:

- Spacer length optimization: Researchers can find ways to adjust the length and flexibility of the spacer connecting the chelating unit and the reporter moiety. This modification helps Schiff base compounds achieve the optimum distance and orientation for efficient cation binding and signal transduction.
- Conformational control: Sensor design should consider incorporating conformational switches within the Schiff base structure. These switches can be responsive to the presence of the target cation, leading to conformational changes that amplify the signal output, thereby improving sensitivity.
- Signal amplification strategies: Introduce amplification mechanisms, such as signal amplification tags, to enhance the signal response generated by the Schiff base structure upon cation binding.
- Analyte-binding pocket modification: Rational design of the binding pocket to optimize interactions with the target cation. This can involve introducing specific binding sites, modifying the cavity size, or incorporating steric hindrance to exclude interfering cations.

## Figures and Tables

**Table 1:** Mechanism, LOD, and applications of the Schiff base probes.

Probe	Analyte	Mechanism	LOD (μM)	Applications	Ref.
1	Hg^2+^	PET/C=N Isomerization/CHEF	0.02	Paper strips, Hg^2+^ recovery from water samples	[66]
2a	Hg^2+^	CHEF	0.27	Hg^2+^ recovery from water samples	[67]
3	Hg^2+^	Metal coordination-spiro ring opening	0.01	HeLa cells, zebrafish	[68]
4a, 4b	Hg^2+^	PET	0.01, 0.01	MCF-7 cells	[69]
5	Hg^2+^	Metal coordination-spiro ring opening	0.03	Cancer (MDA-MB-231) cell imaging	[70]
6	Hg^2+^	CHEF	/	THP-1 cancer cells	[71]
7	Hg^2+^	ICT	0.14	Bovine Serum Albumin, organelle targeting	[72]
8	Hg^2+^	CHEF, aggregation-derived quenching	2	Hg^2+^ recovery from water samples	[73]
9	Hg^2+^	CHEQ	0.023	HeLa cells, paper strips	[50]
10	Cu^2+^	ICT	0.027	/	[74]
11	Cu^2+^	ICT/Metal-induced assembly	0.01	HeLa cells	[75]
12	Cu^2+^	C=N Isomerization/CHEF	0.01	HepG2 Cells, Kunming mouse	[76]
13	Cu^2+^	C=N Isomerization/CHEF	0.0001	HepG2 cells	[77]
14	Cu^2+^	PET	0.003	/	[78]
15	Cu^2+^	CHEF	0.014	Cu^2+^ recovery from water samples	[79]
16	Cu^2+^	C=N Isomerization/CHEF	1.8	/	[80]
17	Cu^2+^, S^2−^	PET	0.015	Paper strips, Cu^2+^ recovery from water and blood samples	[81]
18	Cu^2+^	PET	0.26	RAW 264.7 cells	[82]
19	Cu^2+^	C=N Isomerization/CHEF	1.49	Cu^2+^ recovery from water samples	[83]
20	Cu^2+^	C=N Isomerization/PET	0.49	HepG2 cells	[84]
21	Cu^2+^	C=N Isomerization/ICT/CHEF	0.001	Paper strips, wastewater samples	[85]
22	Fe^2+^	C=N Isomerization/PET	0.036	Fe^2+^ detection in water samples	[86]
23	Fe^3+^	C=N Isomerization	0.038	Cancer cells	[87]
24	Fe^3+^	PET/CHEF	0.014	Fe^3+^ detection in water samples	[88]
25	Fe^2+^, Cu^2+^	PET (Fe^2+^)/MLCT (Cu^2+^)	0.5 (Fe^2+^)	Bovine Serum Albumin	[89]
26	Fe^3+^	C=N Isomerization/PET/LMCT	0.178	Food samples, Fe^3+^ recovery from water samples	[90]
27	Fe^3+^, Al^3+^	TICT	0.172, 0.177	Real water and food samples	[91]
28	Fe^3+^, Cu^2+^	ICT	0.003, 0.002	Zebrafish	[92]
29	Zn^2+^ and Cu^2+^	CHEF	0.18	U251 Glioma Cells	[93]
30	Zn^2+^ and pH	PET	0.056	Zebrafish, live cells	[94]
31	Zn^2+^	ESIPT/CHEF	0.037	HeLa cells, SH-SY5Y neuroblastoma cells	[95]
32	Zn^2+^	PET/CHEF	/	/	[96]
33	Zn^2+^, hydrogen phosphate and cysteine	PET	2.3, 0.21 and 0.16	HeLa cells	[97]
34	Zn^2+^, S^2−^, Fe^3+/2+^	PET/CHEF	1.59, 8.03, 0.73, 1.11	Fe^3+^ tracking in water samples	[98]
35	Zn^2+^	C=N isomerism/CHEF	0.089	HeLa cells	[99]
36	Zn^2+^	PET/ESIPT	0.080	/	[100]
37	Zn^2+^	PET	0.34	Paper strips	[101]
38	Zn^2+^, Cu^2+^, S^2−^	C=N isomerism/ICT	3.6 (Zn^2+^), 0.47 (Cu^2+^)	/	[102]
39	Zn^2+^	PET	0.005	HeLa cells	[103]
40	Zn^2+^	PET	0.0007	B16F10 cell lines 773 and zebrafish	[104]
41	Fe^3+^	ICT/CHEQ	0.036	PC3 cells	[105]
42	Al^3+^	ICT	0.164	HeLa cells, Fe^3+^ recovery from water samples	[106]
43	Cr^3+^	CHEQ	0.041	PC3 cells	[107]

**Table 2:** LOD and applications of Schiff base probes modified with nanomaterials or auxiliary receptors.

Probe	Analyte	Nanomaterial	LOD (μM)	Applications	Ref.
44	Hg^2+^	Gold	0.0006	Hg^2+^ recovery from water samples	[130]
45	Hg^2+^	Silver	0.01	Anticancer, and antibacterial uses	[114]
46	Hg^2+^	Organic	0.008	Hg^2+^ recovery from water samples and living A375 cells	[131]
47	Cu^2+^	Silica/black iron oxide	0.005	Cu^2+^ recovery from water samples	[117]
48	Cu^2+^, Ni^2+^, Fe^2+^	Gold	0.001–0.011	Ni^2+^ tracking in organic waste	[132]
49	Cu^2+^	Polymer	0.243	HeLa cells	[133]
50	Fe^3+^, Cr^2+^, Cd^2+^	Zinc Sulfide	10.24, 31.48, 64.56	/	[134]
51	Pb^2+^	(3-Aminopropyl) triethoxysilane/Gold	0.0001	Paper strips	[135]
52	Hg^2+^	Mesoporous organosilica	/	/	[136]
53	Hg^2+^, Pb^2+^	Graphite	0.0003, 0.001	Hg^2+^and Pb^2+^ tracking in water samples	[137]
54	Cu^2+^	Glass slides	0.015	/	[138]
55	Cu^2+^	Mesoporous silica	0.37	Cu^2+^ extraction from aqueous solutions	[139]
56	Fe^3+^	COFs	/	/	[140]
57	Fe^3+^	Silica cross-linked micellar nanoparticles	0.21	HeLa cells	[141]
58	Fe^3+^, Cu^2+^	Silica	0.44, 0.87	/	[118]
59	Al^3+^	Copper MOFs/Carbon	0.033	Al^3+^ tracking in pharmaceutical and water samples	[142]
60	Zn^2+^	Nanosilica	0.17	/	[143]
61	Zn^2+^	Gold nanoclusters	0.029	Zn^2+^ recovery from fetal bovine serum	[144]
62	Zn^2+^	Gold nanoclusters	0.039	Zn^2+^ recovery from water samples, plasma, urine, and beet root extract; HeLa cells imaging.	[145]
63	Fe^2+^, Zn^2+^	Cd–Ln clusters	/	/	[146]
64	Co^2+^	Lanthanide nanocluster	0.97	/	[147]

## Data Availability

Not applicable.

## Sample Availability

Not available.
